# Deriving a Boolean dynamics to reveal macrophage activation with in vitro temporal cytokine expression profiles

**DOI:** 10.1186/s12859-019-3304-5

**Published:** 2019-12-18

**Authors:** Ricardo Ramirez, Allen Michael Herrera, Joshua Ramirez, Chunjiang Qian, David W. Melton, Paula K. Shireman, Yu-Fang Jin

**Affiliations:** 1 Department of Electrical and Computer Engineering, The University of Texas at San Antonio, 1 UTSA Circle, San Antonio, TX 78249 USA; 2 Department of Anesthesia, Critical Care and Pain Medicine, Beth Israel Deaconess Medical Center, 330 Brookline Ave, Boston, MA 02215 USA; 3 Department of Surgery, Long School of Medicine, University of Texas Health Science Center San Antonio, 7703 Floyd Curl Dr, San Antonio, TX 78229 USA; 4 South Texas Veterans Health Care System, 7400 Merton Minter Blvd, San Antonio, TX 78229 USA

**Keywords:** Macrophage polarization, Boolean networks, Cytokines, Inflammation

## Abstract

**Background:**

Macrophages show versatile functions in innate immunity, infectious diseases, and progression of cancers and cardiovascular diseases. These versatile functions of macrophages are conducted by different macrophage phenotypes classified as classically activated macrophages and alternatively activated macrophages due to different stimuli in the complex in vivo cytokine environment. Dissecting the regulation of macrophage activations will have a significant impact on disease progression and therapeutic strategy. Mathematical modeling of macrophage activation can improve the understanding of this biological process through quantitative analysis and provide guidance to facilitate future experimental design. However, few results have been reported for a complete model of macrophage activation patterns.

**Results:**

We globally searched and reviewed literature for macrophage activation from PubMed databases and screened the published experimental results. Temporal in vitro macrophage cytokine expression profiles from published results were selected to establish Boolean network models for macrophage activation patterns in response to three different stimuli. A combination of modeling methods including clustering, binarization, linear programming (LP), Boolean function determination, and semi-tensor product was applied to establish Boolean networks to quantify three macrophage activation patterns. The structure of the networks was confirmed based on protein-protein-interaction databases, pathway databases, and published experimental results. Computational predictions of the network evolution were compared against real experimental results to validate the effectiveness of the Boolean network models.

**Conclusion:**

Three macrophage activation core evolution maps were established based on the Boolean networks using Matlab. Cytokine signatures of macrophage activation patterns were identified, providing a possible determination of macrophage activations using extracellular cytokine measurements.

## Background

Over the past 30 years, extensive research has been dedicated to investigating the role of macrophages due to its versatile functions in innate immunity, infectious diseases, and progression of cancers and cardiovascular diseases, the top 2 leading causes of death in the world [1–4]. Macrophages have bactericidal and phagocytic functions and regulate immune responses and the development of inflammation by secreting monokines including enzymes, complement proteins, and regulatory factors such as IL-1 (Interleukin-1), IL-6, and IL-12 [5]. M2 macrophages turn off the damaging immune system activation, function in constructive processes like wound healing and tissue repair, and coordinate the chronic inflammatory response by regulating downstream cellular functions (inflammation resolution, endothelial cell, and fibroblast activation) [4, 6–8]. These contradictory functions of macrophages were conducted by different macrophage phenotypes classified as M1 (classically activated macrophages) and M2 (alternatively activated macrophages) due to different stimuli in the complex in vivo cytokine environment.

Macrophages have shown in vitro M1 activation with Lipopolysaccharide (LPS), interferon-gamma (IFN-γ), or TNF (Tissue Necrotic Factor) stimuli. M1 macrophages secrete high levels of IL-12 and IL-23 and low levels of IL-10. In contrast, M2 macrophages secrete high levels of IL-10, Transforming Growth Factor–β (TGF-β) and low levels of IL-12 and IL-23. Recently, M2 activation has been further classified into 4 sub-phenotypes, M2a, M2b, M2c, and M2d. M2a activation is stimulated by interleukin-4 (IL-4) and IL-13, while M2b with immune complex (IC) + Toll-like receptor (TLR), IC + IL-1 receptor (IL1R), or IL-1β, M2c with IL-10 or TGF-β stimuli, and M2d with TLR ligands or adenosine receptor ligands [9–12]. In addition, switches between M1 and M2 phenotypes have been reported [13–15].

Analysis of macrophage activations has been reported by others and our group. We have reported differential equation models on macrophage activation and effects of matrix metalloproteinase-9, and − 28 on macrophage activations [16–18]. Martin and colleagues have validated a Boolean dynamics of genetic regulatory network for LPS-stimulated macrophage activation using temporal microarray (mRNA) data at 8-time points (15mins, 30mins, 1 h, 2 h, 4 h, 8 h, 16 h, 32 h) [19]. Rex’s group characterized the inflammatory gene expression patterns of LPS-stimulated M1 activation and IL-4 and IL-13-stimulated M2 activation at 0.5, 1, 2, 6, 10, and 24 h, and modeled macrophage activations by combining Boolean dynamics and differential equations [20]. Though less than 50 genes were considered in each macrophage activation model, the limited 6 temporal measurements still led to an unavoidable difficulty in parameter estimation for differential equation models. Recently, transcriptional and post-transcriptional graphical networks of macrophage in healthy and diseased hearts were reported [21]. All these models focused on the regulatory networks at the gene level and gave a deeper insight of what happened inside a cell. Interestingly, all models reported significant cytokine markers for macrophage activations. Furthermore, in an in vivo situation, stimuli and final secretion products of activated macrophages stay outside the cells. It’s natural and practical to define macrophage phenotypes with cytokine expressions for an Input-Output representation only based on cytokine expressions outside of the cells. Therefore, in vitro cytokine profiles from macrophage activations serve perfectly to explore such abstract models.

We globally searched “temporal” or “time series” and “macrophage activation” or “macrophage polarization” in PubMed, GEO datasets/profiles, and ProteomeExchange to screen specific macrophage activation temporal profiles with different stimuli. A total of 173 datasets were found for *Mus musculus* and 156 for *Homo sapiens* as of June 18, 2019. Eleven studies focused on temporal macrophage activations. Currently, complete temporal in vivo genome or proteome expression profiles of macrophage activation has not been reported. However, in vitro temporal data has been deposited in public databases to shed a light on macrophage activation mechanisms. A dataset from Melton’s group was selected to establish a Boolean model because macrophage activation expression profiles of 27 cytokines for 4 groups (a control group and 3 activation groups with 3 different stimuli, LPS, IL-4, and IL-10) at 7 points (0 h, 0.5 h, 1 h, 3 h, 6 h, 12 h, 24 h) were documented [22]. This dataset provides extra information on time points, sub-activation groups, and cytokine expression levels to existing models on gene regulations [19–21].

In this study, expression levels of the selected differentially expressed cytokines were binarized with “1” (up-regulated) and “0” (down-regulated). Linear programming algorithm was applied to determine the structure (links and interaction strengths) of a regulatory network for macrophage activations among the 27 selected cytokines under 3 different stimuli. Based on the obtained network structure, Boolean functions were determined for each cytokine in the network using a Karnaugh Map. For the first time, core networks for macrophage activation models were generated and mathematically represented with the semi-tensor product. The core network for M1, M2a, and M2c only contained 6, 7, and 5 proteins, respectively. Links of macrophage activation networks were validated based on public Protein-Protein Interaction (PPI) and Kegg pathway databases. The core network for each activation pattern was validated by the coincidence of predicted and temporal experimental results. A novel temporal evolution map for each macrophage activation model was also generated to show all Boolean states, suggesting possible evolutionary paths of the networks with different initial conditions.

## Results

### Curve fitting with smooth spline algorithm

**Fig. 1:**
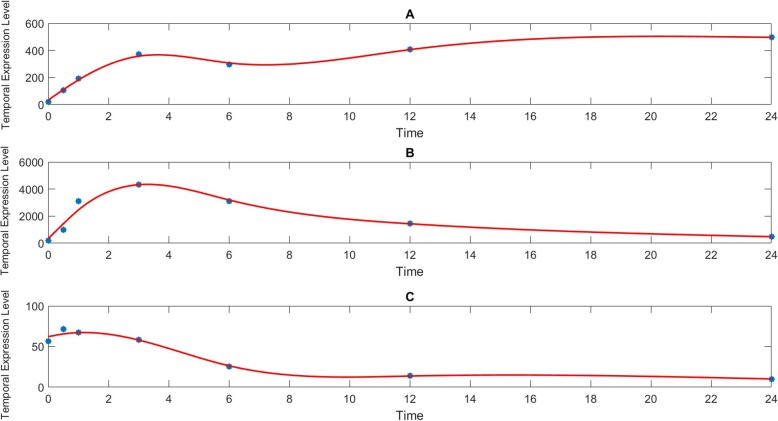
Three typical temporal protein expression profiles for macrophage activations. Horizontal coordinate represents time and the vertical coordinate denotes expression levels of proteins. The red line shows the constructed continuous profile while the blue stars denote the real experimental data at each sampling time point. **a** The profile of MCP-5 in M2c activation shows a promotion pattern. **b** The profile of MCP-3 in M1 activation shows a bell curve. **c** The profile of IP-10 in M2a activation shows an inhibition pattern

An illustration of 3 typical expression profiles by curve fitting was shown in Fig. 1. The 3 proteins are C-C motif chemokines ligand 12 (CCL12, also named as MCP-5) in M2c activation, CCL7 (MCP-3) in M1 activation, and chemokine (C-X-C motif) ligand 10 (CXCL10, also named as IP-10) in M2a activation. Interestingly, MCP-5 followed a similar profile as promotion, ($c\frac{t}{1+t}\Big)$, where *c* represents a constant of its max amplitude with respect to time *t* (Fig. 1a). MCP-3 followed a bell shape curve similar to a Gaussian distribution in Fig. 1b, and IP-10 followed an inhibition relatable to, ($c\frac{1}{1+t}+ bias\Big)$, in Fig. 1c. The *bias* denotes an offset term since an expression may not necessarily reach 0. The average R-Squared error of the fitting algorithm was 0.9087, 0.8566, and 0.8106 for M1, M2a, and M2c group, respectively.

A temporal profile is considered as a promotion if its final expression level is more than twice or inhibition if it is less than half of the initial expression level, and a bell-curve otherwise. It’s worth to mention that an expression profile demonstrates different patterns with different time periods due to activation dynamics. About 138 out of 324 (27x4x3, 27 protein expression measured from 4 replicates in 3 activation groups) protein expression profiles followed a promotion pattern, 27 had inhibition profiles, and 148 had a bell curve within the first 6 h as shown in Table 1. Examining the complete 24 h time span, 78 profiles fit a promotion, 110 fit an inhibition while 125 for a bell curve, suggesting significant protein up-regulation in the first 6 h and gradually evolve to stable polarizations. M2 activation profiles demonstrate more inhibition patterns compared to M1 activation in Table 1. Chemokine (C-X-C motif) ligand 1 (CXCL1/KC-GRO) did not or barely reached detectable levels in both M2a and M2c activations. These profiles were denoted as N/A in Table 1. Therefore, KC-GRO was not considered in either M2 activation models for undetectable expression levels. GM-CSF (Granulocyte-macrophage colony-stimulating factor) from some replicates reached a detectable level 12 h post-stimulation in M2a and M2c and was included in both M2 models. KC-GRO was significantly up-regulated in M1 activation, illustrating their role to identify M1 activation [23]. All other protein expression levels are either elevated or down-regulated comparing against the average of the control group at least 1 out of 7 time points with *p*-value < 0.05.

**Table 1:** Properties of Expression Profiles

Activation	Time	Bell Curve	Promotion	Inhibition	N/A	Total
M1	6 h	26	74	8	0	108
	9 h	36	61	11	0	108
	24 h	33	46	29	0	108
M2a	6 h	60	29	13	6	108
	9 h	57	17	28	6	108
	24 h	57	3	42	6	108
M2c	6 h	62	35	6	5	108
	9 h	64	32	7	5	108
	24 h	35	29	39	5	108
Total	6 h	148	138	27	11	324
	9 h	157	110	46	11	324
	24 h	125	78	110	11	324

### Binarization

**Fig. 2:**
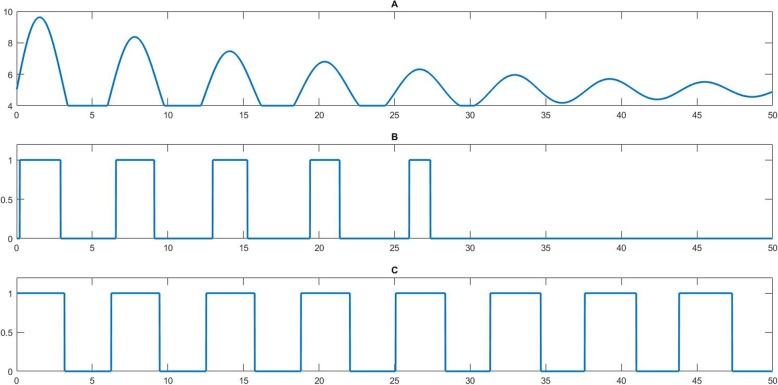
Comparison of binarization threshold using K-means and iterative K-means methods. Analyzing the same expression profiles in (**a**), the K-means method identified the first 5 peaks with relatively high expression levels in (**b**) while the iterative K-means method identified all peaks in (**c**)

Both normal K-means and iterative K-means algorithms were applied to the 50 re-sampled data from the fitted curves. Figure 2 gives an illustration of 2 clusters with the normal K-means method and the final binarization of the iterative K-means method. The Iterative K-means generates better binarization results than the normal K-means method since it identifies more significant peaks in the conversion.

**Fig. 3:**
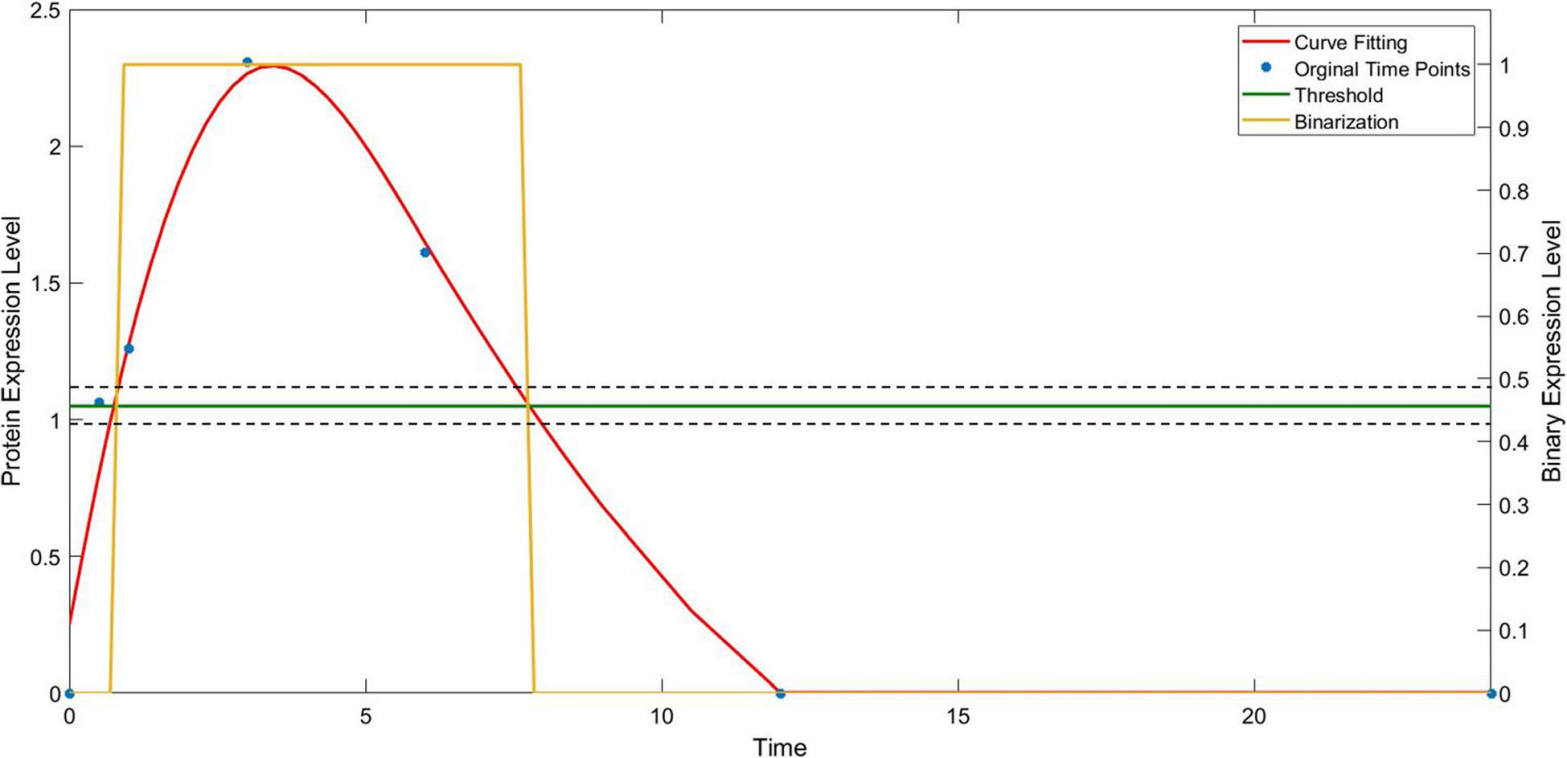
Binarization of a protein expression level. The blue stars show the experimental measurements, the red curve is the fitted continuous profile, the green line represents the threshold from the iterative k means method, and the orange line denotes the final binarization. An illustration of a possible sensitivity region is shown between the two dashed black lines as an error band

A binarization obtained from the smooth spline and iterative K-means is shown in Fig. 3. The blue stars represent the initial time points (0 h, 0.5 h, 1 h, 3 h, 6 h, 12 h, 24 h) in the dataset, the red solid line represents the curve fitting result, the green solid line gives the threshold from the iterative K-means, and the yellow solid line is the final binarization result. An error band centered at the binarization threshold is denoted by dashed lines for possible binarization errors.

### Statistical analysis

All experimental measurements at 7 sample times have been analyzed in Melton’s paper for statistical significance [22]. We confirmed their analysis with the Kolmogorov-Smirnov test (*p* < 0.01). We fitted temporal expression profiles for all genes with detectable expression levels in 3 stimulated groups and 1 control group. Two-sample Kolmogorov-Smirnov tests were performed on fitted temporal profiles of each gene given a stimulus versus the control with a significance value *p* < 0.01.

The fitted smooth spline functions are determined by minimizing the summation of squared errors at seven time points. The average error at these seven sample time points can be obtained in Matlab. Such error may lead to a false determination of binarization value. To test the sensitivity of binarization, an error band was formed as a binarization threshold ± error. If any of the 50 re-sampled data from the averaged fitted expression stays in the error band, there might be a false binarization and the re-sampled data is sensitive to binarization. The maximum likelihood for false binarization is less than 5% for all genes in 3 stimuli groups. No outlier is detected in our experimental and fitted data.

### Determining Boolean functions

There are three assumptions in determining the Boolean functions of a network model. 1) Significant functional changes appear in a period larger than the simulation intervals; 2) The cycles of macrophage activation are larger than our simulation interval; 3) The Boolean network will have a fixed structure and regulation during the 24-h response period in the experiment.

Since macrophage activations have the most significant changes in the first 9 h post-stimulation, we put 40 samples during the first 9 h, which led to a simulation interval of about 13.5 min. The other 10 out of 50 samples were evenly re-sampled from 9 to 24 h. Therefore, the simulation interval is 13.5 min in this study.

Currently, most of the published results show a period of 24 h for macrophages’ responses to stimuli. In addition, experimental data are collected with different time periods ranging from 15 min to hours [19–21, 24]. Our simulation time interval is very similar to the minimum sampling time of available experimental measurements (15 min). This validated assumptions 1 and 2. Since biological regulations have demonstrated stability and robustness, assumption 3 is a general assumption for modeling.

Boolean functions are determined with respect to binarized values in a time period instead of a single distinct time point. We verified the Boolean function determined by the averaged gene expressions with two methods: agreement with Boolean function dependent on experimental results and agreement of promotion/inhibition determined by Linear Programming. A total of 23 out of 27 datasets (85%) have shown the agreement of Boolean functions determined by the average expressions of genes from 4 replicates with a total of 50 samples for 24 h post-stimuli and linear programming results.

**Fig. 4:**
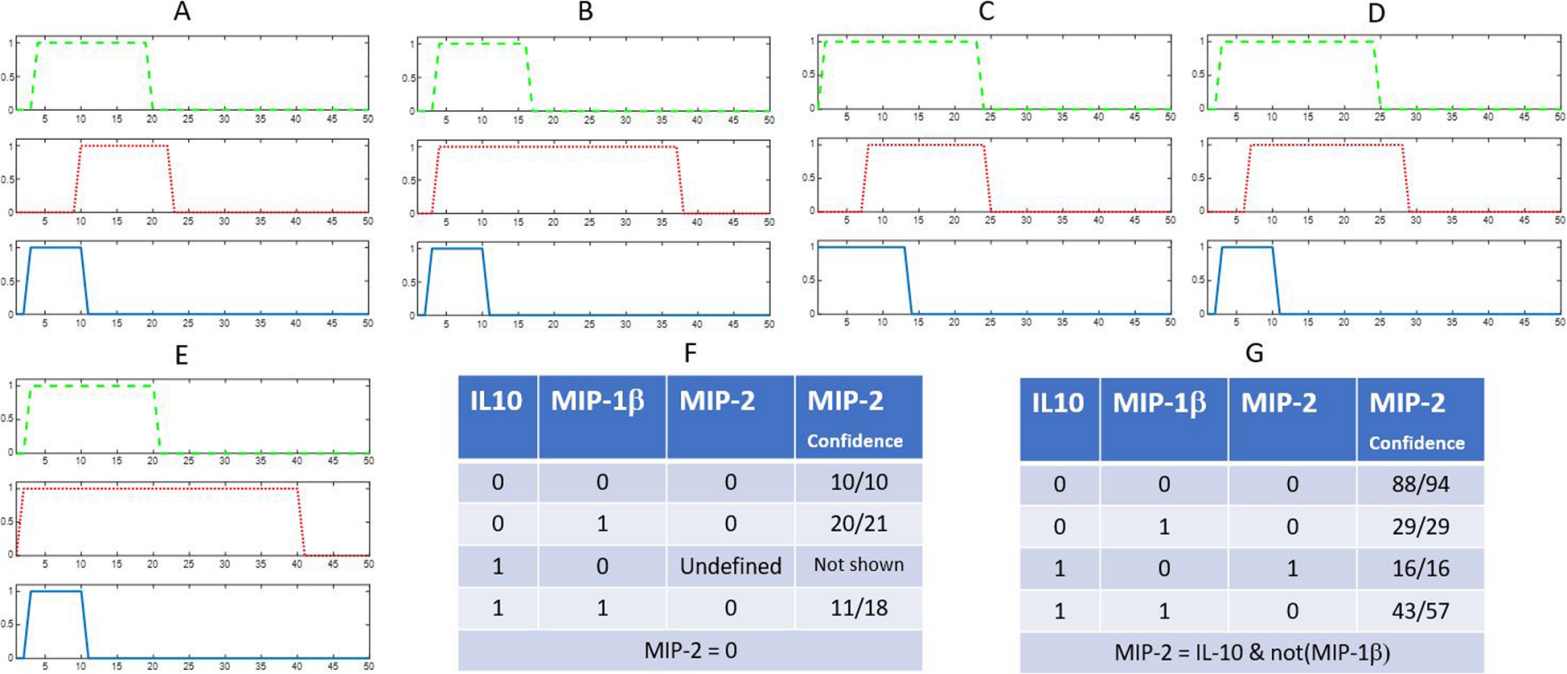
The Binary Expressions of a simple network with two inputs (MIP-1β and IL10) and one output (MIP-2). The sub-Figs. **a**-**d** showed the binarization of 4 replicates of the LPS stimulated responses and **e** being the binarization of the average of 4 replicates. The green dashed line represents IL-10, the red dotted for MIP-1β and the blue solid line for MIP-2. The Karnaugh maps for the average and each replicate are shown in **f** and **g** respectively

However, variations of temporal responses exist in biological systems [25]. The variations may be more severe during the transition between two different states. If there is any disagreement of Boolean function determined by Karnaugh map and experimental measurement or Linear programming results, we further determined the Boolean function among proteins with respect to each individual experimental replicate instead of the average of 4 replicates and enriched our samples to be 200. An illustrative example showing the agreement of Boolean functions determined by the averaged expression and individual replicates is shown in Fig. 4. In a small network with two inputs (IL-10 and MIP-1β) and one output (MIP-2), the averaged profiles have a missing state (IL-10 = 1 and MIP-1β = 0), so the Boolean function determined by the Karnaugh map showed MIP-2 = 0, contradicting to our experimental results. In this case, expression levels of 4 replicates are considered together to determine the Boolean function. Note that there are only 49 (average) and 196 (4 replicates) sample time points for the iterative evolution ends at sample 49 to predict the state at sample time point 50. The agreement of this Boolean function is about 90%.

### Properties of the networks established with linear programming algorithm

Linear programming algorithm was applied to determine the network structure for 3 macrophage activation patterns. As shown in Table 2, the networks obtained were still complex. To simplify the network structure, a link between two nodes was removed if its weight calculated by linear programming was less than 70% of the maximum link weight described in the Method Section. The 70% threshold was chosen based on the criteria to minimize the number of links and maximize the summation of link weights. Properties of the networks established with original linear programming and the simplified network were shown in Table 2. Input nodes are proteins with no parent nodes and output nodes are proteins with no child nodes. All 3 simplified networks have fewer nodes and links than the original results obtained from the linear programming algorithm. In addition, the complexities of M1 and M2a polarization networks are less than the networks for M2c polarization, specifically for the number of links and network density. While the M2c activation network has 3 nodes with ≥4 parent nodes, these 3 nodes are all output nodes and will not affect the regulation of Boolean functions, suggesting that the 50 sample data should be sufficient to establish the Boolean functions.

**Table 2:** Properties of M1, M2a, and M2c Activation Networks

	M1	M1-simplified	M2a	M2a- simplified	M2c	M2c- simplified
Number of Nodes	27	26	27	24	27	24
Network Density	12.9	1.5	12.9	1.5	15	1.75
Number of Links	348	39	348	36	404	42
Number of Promotion links	189	25	189	19	211	25
Number of Inhibition links	159	14	159	17	193	17
Number of Input Nodes	0	2	0	1	0	0
Number of Output Nodes	5	10	6	11	5	9
Number of Attractor	N/A	1	N/A	0	N/A	0
Nodes with ≥4 Inputs	N/A	0	N/A	0	N/A	3

### LPS induced M1 activation network

**Fig. 5:**
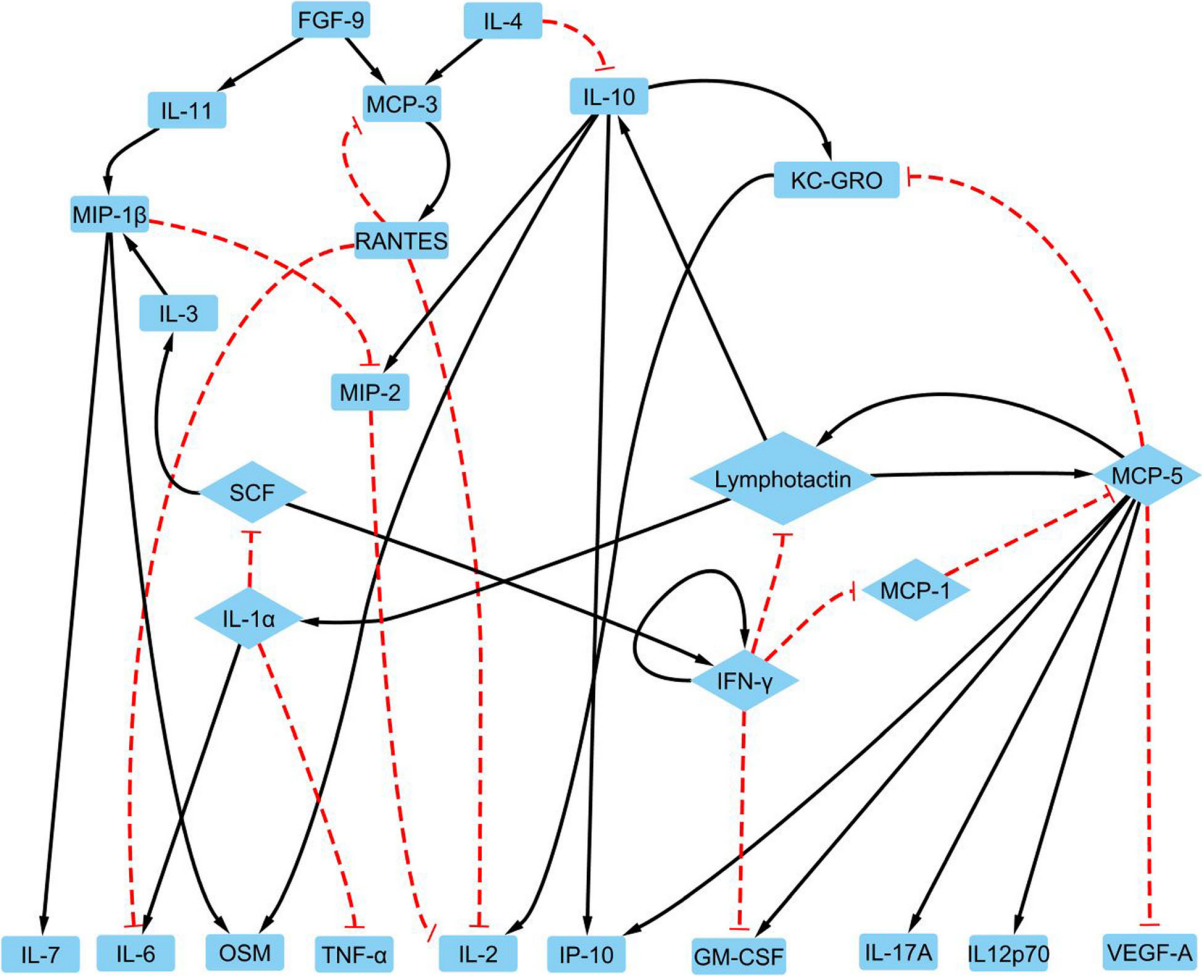
The network for M1 activation includes 26 nodes with each node representing a protein (blue block), a black line with an arrow for a positive connection from a parent node to a child node, and logic negation being a red dashed connection with a ‘T’ arrow. The core network is shown as the diamond nodes in the network

The structure for the LPS induced M1 activation network was shown in Fig. 5. Boolean regulations for each node were shown in Table 3. An overbar denotes a negation, while a double vertical bar for a logic ‘OR’ and a ‘&’ symbol representing a logic ‘AND’. In addition, interaction strengths calculated by the linear programming algorithm for each link were listed in Table 3.

**Table 3:** Boolean Functions and Description of LPS Stimulated M1 Activation Network. IS: intermediate state

Child node	Parent node	Validation	Attribution	Weights from LP
FGF-9(K + 1)	N/A	Input- [26]	Input	N/A
GM-CSF(K + 1)	$\overline{\mathrm{IFN}-\mathrm{y}}$ (K) & MCP-5(K)	IFN- γ: String [27], MCP-5 [28–30]:	Output	(− 180, 185)
KC-GRO(K + 1)	IL-10(K)&$\overline{\mathrm{MCP}-5}$ (K)	IL-10: String MCP-5: String	IS	(5.45, −5.82)
IFN-γ(K + 1)	SCF(K) || IFN- γ (K)	IFN-γ [31, 32]: SCF [33, 34]:	IS	(1170)
IP-10(K + 1)	IL-10(K)& MCP-5 (K)	MCP-5: String [33, 35, 36], IL-10 [33, 35, 37] [38]:	Output	(68,100, 85,000)
IL-1α(K + 1)	Lymphotactin(K)	KEGG	IS	(787000)
IL-2(K + 1)	($\overline{\mathrm{KC}-\mathrm{GRO}}$ (K)&$\overline{\mathrm{MIP}-2}$ K)) || (KC-GRO(K)&$\overline{\mathrm{Rantes}}$ (K))	KC-GRO: String [39, 40], MIP-2: String [41], RANTES: [33, 42, 43]	Output	(34.7,-27, − 33.8)
IL-3(K + 1)	SCF(K)	SCF: String	IS	(302)
IL-4(K + 1)	N/A	Input [44]:	Input	N/A
IL-6(K + 1)	IL-1α(K) &$\overline{\mathrm{Rantes}}$ (K)	IL-1α: String [45, 46], RANTES: String	Output	(3840, − 4840)
IL-7(K + 1)	MIP-1β(K)	MIP-1β: String [47],	Output	(10)
IL-10(K + 1)	$\overline{\mathrm{IL}-4}$ (K) &Lymphotactin(K)	IL-4: String [48] [49], Lymphotactin: String	IS	(− 64,700, 48,600)
IL-11(K + 1)	FGF-9 (K)	FGF-9-String	IS	(6190)
IL-12p70(K + 1)	MCP-5(K)	KEGG	Output	5.6
IL-17A (K + 1)	MCP-5(K)	KEGG	Output	(0.555)
Lympho- tactin(K + 1)	$\overline{\mathrm{IFN}-\mathrm{y}}$ (K)	IFN- γ: String [50],	IS	− 1675
MIP-1β(K + 1)	IL-3(K) || IL-11(K))	IL-3: String IL-11: String	IS	(44,800, 41,600)
MIP-2(K + 1)	IL-10(K)& $\overline{\mathrm{MIP}-1\upbeta}$ (K)	IL-10: String [51] MIP-1β: String [52]	IS	(7880, − 10,400)
MCP-1(K + 1)	$\overline{\mathrm{IFN}-\mathrm{y}}$ (K)	IFN- γ: String [53]	IS	(− 5090)
MCP-3(K + 1)	(IL-4(K)& (FGF-9(K))|| ($\overline{\mathrm{FGF}-9}$(K) & $\overline{\mathrm{Rantes}}$ (K)))	FGF-9 [26]: IL-4 [54]: Rantes: String	IS	(27,600, 22,800, − 27,400)
MCP-5(K + 1)	Lymphotactin(K) &MCP-1(K)	Lymphotactin: String	IS	(5840, − 4590)
OSM(K + 1)	IL-10(K) & MIP-1β(K)	IL-10 [55]: MIP-1β: String	Output	(6.45, 6.19)
SCF(K + 1)	$\overline{\mathrm{IL}-1\upalpha}$ (K)	IL-1α [56]:	IS	(− 70,800)
RANTES(K + 1)	$\overline{\mathrm{MCP}-3}$ (K)	KEGG	IS	(304)
TNF-α(K + 1)	$\overline{\mathrm{IL}-1\upalpha}$ (K)	IL-1α: String [57]	Output	(− 1.37)
VEGF-A(K + 1)	$\overline{\mathrm{MCP}-5}$ (K)	MCP-5 [58]:	Output	(− 17,850)

There are 26 proteins in this network. TIMP-1 (Tissue Inhibitor of Metalloproteinases) is the only protein removed from the 27 measured proteins as an orphan node. The two input nodes are FGF-9 (Fibroblast Growth Factor-9) and IL-4. FGF-9 has been known to be an inflammation promoter in multiple sclerosis [26]. Interestingly, IL-4, not LPS, was annotated as input for the M1 activation network. Though IL-4 is known as a stimuli for M2a activation and anti-inflammatory responses, the combination of LPS and IL-4 has been reported for its promotion of other inflammation cytokines including IL-6, CCL-1, CCL-3 (macrophage inflammatory protein 1-α, MIP-1α), CCL-4 (macrophage inflammatory protein 1-β, MIP-1β), CCL-5 (Regulated upon Activation, Normal T cell Expressed, and Secreted, RANTES), TNF-α, and IFN-γ, which are also included in our M1 activation network [44]. In addition, LPS has been shown to induce IL-4 gene expression, which confirmed the rationale for IL-4 serving as an input for the M1 activation network [59]. The M1 activation network has 10 output nodes: GM-CSF, IL-2, IL-6, IL-7, IL-12p70, IL-17A, IP-10, OSM (Oncostatin M), TNF-α, and VEGF-A (Vascular Endothelial Growth Factor A). All these proteins’ expressions are significantly upregulated compared to the control group.

### Validation of LPS induced M1 activation network model

To further validate the links in our network model, Protein-Protein-Interaction (PPI) database (STRING), KEGG pathway database, and literature results were used to confirm interactions among proteins in the network. The majority of links (35 out of 39) were confirmed by STRING directly. The other 4 links can be verified through the KEGG pathway database as shown in Table 3. Lymphotactin can trigger the chemokine receptor pathway which can further stimulate multiple pathways such as Salmonella infection to produce IL-1α. MCP-5 to IL-12p70 link can be verified through MCP-5, Chemokine receptor pathway, MAPK signaling pathway, and then RIG-I-like receptor signaling pathway that leads to the synthesis of inflammatory cytokines including Il-12. Similarly, MCP-5 can also trigger the secretion of IL-17 through chemokine receptor pathway, JAK-STAT signaling pathway, and Th17 cell differentiation pathway. MCP-3 to RANTES link can possibly be verified through MCP-5, Chemokine Receptor Pathway, MAPK signaling pathways, Toll-like receptor signaling pathway and Herpes simplex virus 1 infection/ Influenza A pathway.

There were 5 feedback loops as shown in Fig. 5: 1) IFN-γ attractor; 2) from IFN-γ to itself through MCP-1 (monocyte chemoattractant protein), MCP-5, Lymphotactin, IL-1α, and SCF (Stem Cell Factor); 3) from IFN-γ to itself through Lymphotactin, IL-1α, and SCF; 4) a loop between Lymphotactin and MCP-5, and 5) a loop between MCP-3 and RANTES. Interestingly, the first three loops illustrated an IFN-γ attractor self-promotion loop during macrophage activation [60]. STRING database showed MCP-3 and RANTES, MCP-5 and Lymphotactin are interaction pairs.

Figure 5 showed the complete Boolean network generated with our temporal profiles. The 10 output nodes (from the leftmost node IL-7 to the rightmost node VEGF-A at the bottom of Fig. 5) do not affect the regulations of the network and their states can be determined by their parent nodes. In addition, the signal flows from FGF-9 to IL-11 to MIP-1β and from SCF to IL-3 to MIP-1β form single direction forward links, therefore, given the status of input node FGF-9, the status of MIP-1β can be determined together with SCF. Similarly, signal flow from IL-4 to IL-10 to KC-GRO forms single directional links. The input node IL-4 and Lymphotactin expression levels determine IL-10 and KC-GRO expression levels, suggesting Lymphotactin should be a major in evolution and IL-10 and KC-GRO are dependent variables in the simulations and can be removed. Therefore, removing output and single direction transduction nodes led to a simplified network with only 8 proteins for two sub-networks: MCP-3 – RANTES loop for sub-network 1 and IFN-γ, MCP-1, MCP-5, Lymphotactin, IL-1α, and stem cell factor (SCF) for a core 6-protein (in diamond shapes) network as shown in Fig. 5. States of other nodes in the M1 activation network can be determined by the states of these 8 proteins and the input nodes.

**Fig. 6:**
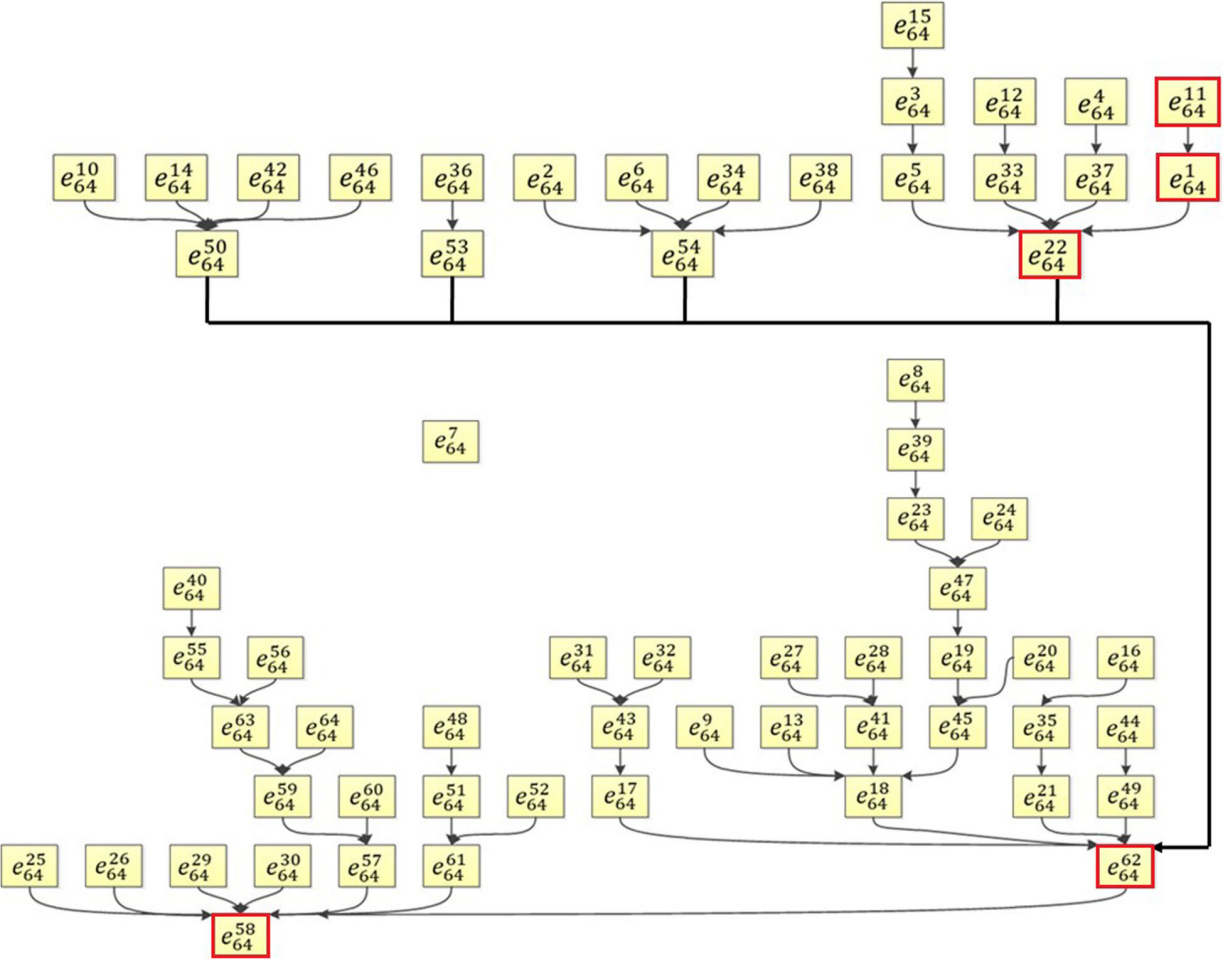
The evolution map of the core network for M1 activation showed all possible pathways predicted by the M1 Boolean network. The evolution of 64 (64 = 2^6^) states for 6 nodes in the core network were illustrated from different initial conditions. The states in red boxes represent the evolution path based on the temporal cytokine profiles in this study

**Fig. 7:**
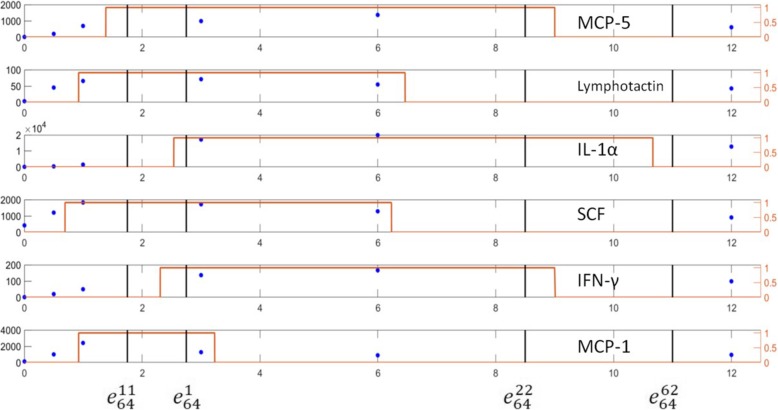
Experimental expression levels of the 6 proteins in the core network for M1 activation were compared with their binary profiles. The blue stars showed the measured experimental expression levels at 6 time points (0, 0.5 h, 1 h, 3 h, 6 h, and 12 h) while the orange line illustrated the temporal binary expression. The vertical black bars represent the states that are in our M1 activation evolution map

The state transition map for the M1 activation core network was shown in Fig. 6. The state transition map contains 64 = 2^6^ states with one isolate state ${e}_{64}^7$ and the final state ${e}_{64}^{58}$. The state ${e}_{64}^{62}$ is an important transition state with multiple incoming branches. Our binarized and original temporal profiles showed the transition from ${e}_{64}^{11}\to {e}_{64}^1\to {e}_{64}^{22}\to {e}_{64}^{62}\to {e}_{64}^{58}$ and these states were illustrated in red boxes in Fig. 6. The binary values of these 5 transition states were shown in Table 4. The state numbers are illustrated in two’s complement representation. Temporal transitions for the 6 proteins with binary states and sampled data were shown in Fig. 7.

**Table 4:** Binary Expressions of Proteins in the Core Network for LPS Stimulated Macrophage Activation. The binarization is shown in two’s complement representation

State	MCP-5	Lymphotactin	IL-1α	SCF	IFN- γ	MCP-1
${e}_{64}^{11}$	1	1	0	1	0	1
${e}_{64}^1$	1	1	1	1	1	1
${e}_{64}^{22}$	1	0	1	0	1	0
${e}_{64}^{62}$	0	0	0	0	1	0
${e}_{64}^{58}$	0	0	0	1	1	0

It’s worth to mention, not all 4 experiments for M1 activation converged at the same time. One replication of this experiment reached the state ${e}_{64}^{58}$ while the other 3 ended at the state ${e}_{64}^{62}$. The coincidence of state transition from experimental results and computational results demonstrated the effectiveness of the core network model of M1 activation.

### M2 activation networks

**Fig. 8:**
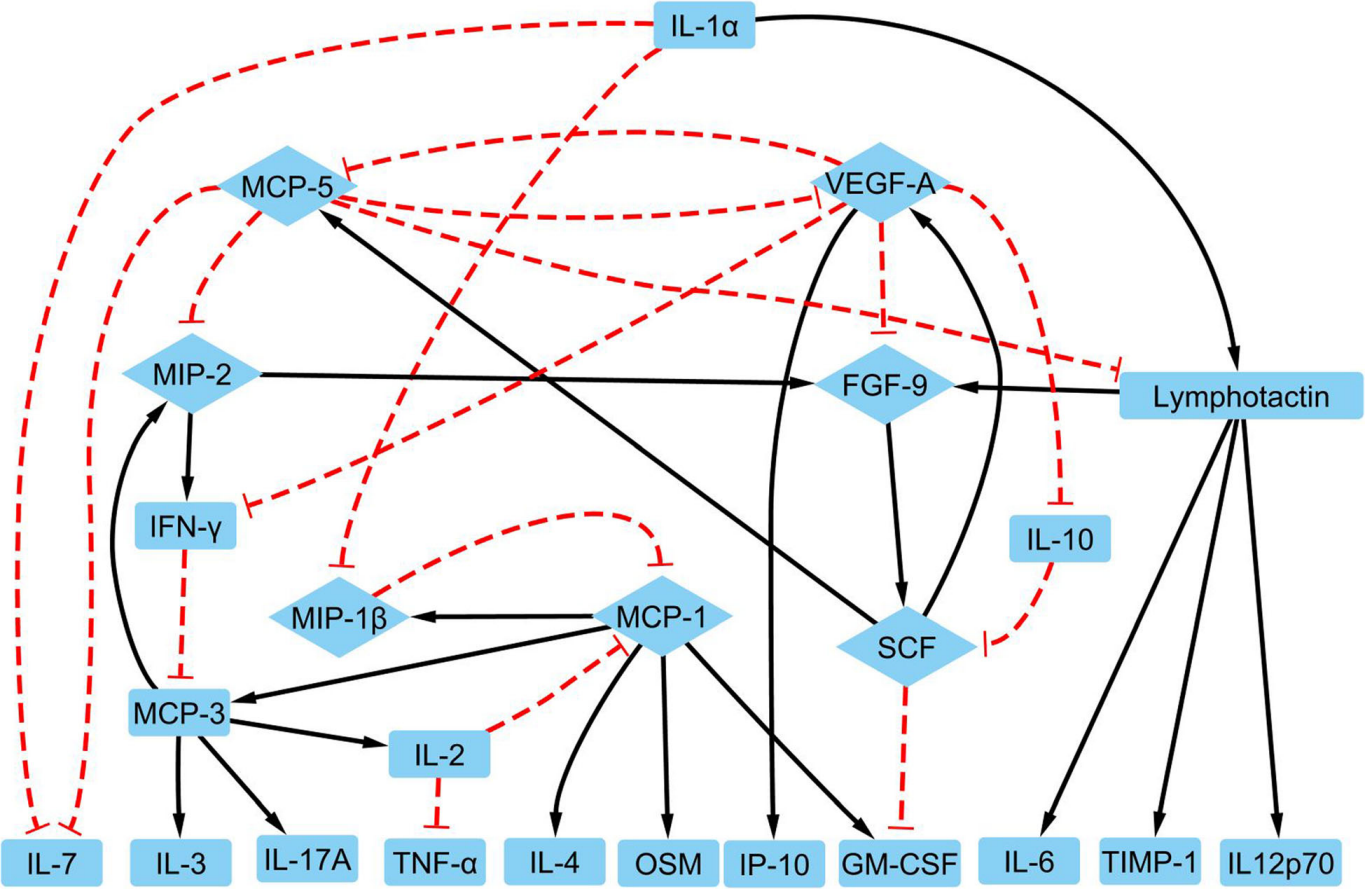
The network for M2a activation includes 24 nodes with each node representing a protein (blue block), a black line with an arrow for a positive connection from a parent node to a child node, and logic negation being a red dashed connection with a ‘T’ arrow. The core network is shown as the diamond nodes in the network

**Fig. 9:**
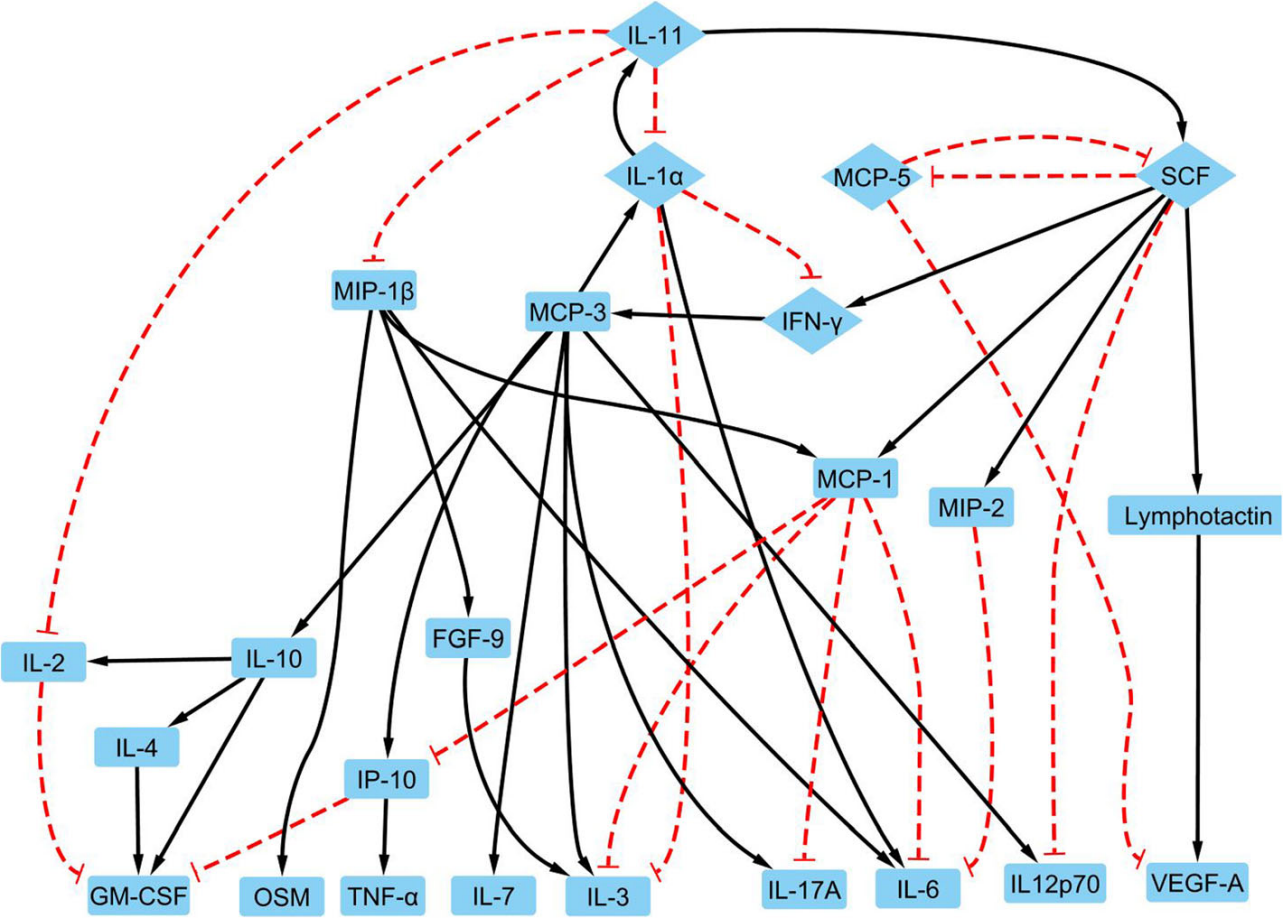
The network for M2c activation includes 24 nodes with each node representing a protein (blue block), a black line with an arrow for a positive connection from a parent node to a child node, and logic negation being a red dashed connection with a ‘T’ arrow. The core network is shown as the diamond nodes in the network

Boolean networks for IL-4 induced M2a and IL-10 induced M2c activations have been established and shown in Fig. 8 and Fig. 9, respectively. M2 activations were more complicated than M1 activation due to variant stimuli and M2 subtypes [11, 14]. These were represented by the increased network density and number of links in the M2c activation network compared against the M1 activation network as shown in Table 2.

Though M2a and M2c networks shared common responses, different subtypes also illustrated specific responses [3]. In the M2a and M2c activation networks, there were 23 common nodes as shown in Tables 5 and 6. The only two different proteins between M2a and M2c are TIMP-1 and IL-11. TIMP-1 was included in both M2a and M2c networks from our linear programming results. However, TIMP-1 was an orphan node in the M2c network and not shown in Table 6 and Fig. 9. Previous results have shown that TIMP-1 is consistently produced by macrophages and further induced by LPS (M1) and IL-10 (M2c) in fully differentiated macrophages [128, 129]. Additionally, TIMP-1 was down-regulated by IL-4 in M2a [130]. Since TIMP-1 functions by forming one-to-one complexes with target metalloproteinases and there is no measurement on metalloproteinases in this study, TIMP-1 is classified as an orphan node. IL-11 is included in the M2c but not the M2a network in this study. IL-11 is a member of the IL-6-type cytokine family and may provide a link between inflammation and cancer [131, 132]. The role of IL-11 in macrophage activation has not been well studied yet.

**Table 5:** Boolean Functions and Description of IL-4 Stimulated M2a Activation Network. The * symbol denotes a non-validated link from public databases or literature

Child node	Parent node	Validation	Attribution	LP Weights
FGF-9(K + 1)	($\overline{\mathrm{MIP}-2}$ *(k)&$\overline{\mathrm{Vegf}-\mathrm{A}}$ (k)) || (MIP-2(k) & VEGF-A(k)) || ($\overline{\mathrm{MIP}-2}$ (k) &Lymphotactin(k))	VEGF-A: String [61] [62, 63], Lymphotactin [64]:	IS	(89.6, 87, − 82)
GM-CSF(K + 1)	$\overline{\mathrm{MCP}-1}$ (K) || SCF(K)	MCP-1: String [65, 66], SCF: String	Output	(2.75, −3.23)
IFN- γ (K + 1)	MIP-2(k) || $\overline{\mathrm{Vegf}-\mathrm{A}}$ (k)	MIP-2: String [67, 68], VEGF-A: String	IS	(34, − 31.7)
IP-10(K + 1)	VEGF-A(K)	[69, 70]	IS	121
IL-1α(K + 1)	N/A	Input [71]:	Input	N/A
IL-2(K + 1)	MCP-3(K)	String	IS	92.7
IL-3(K + 1)	MCP-3(K)	[72, 73]	Output	41.9
IL-4(K + 1)	MCP-1(K)	String [74–76],	Output	151
IL-6(K + 1)	Lymphotactin(K)	[77]	Output	58.7
IL-7(K + 1)	IL-1α(K) & MCP-5* (K)	IL-1α: [78]	Output	(−0.9, − 1)
IL-10(K + 1)	VEGF-A(K)	[79]	IS	− 296
IL-12p70(K + 1)	Lymphotactin(K)	KEGG	Output	0.557
IL-17A(K + 1)	MCP-3(K)	MCP-3 [80, 81]:	Output	(.34)
Lympho-tactin(K + 1)	IL-1α (K) & MCP-5 (K)	[82]	IS	(192, − 159)
MIP-1ϐ(K + 1)	IL-1α(K) & MCP-1(K))	IL-1α: String [83] [84] MCP-1: String, [85] [86]	IS	(− 6490, 6650)
MIP-2(K + 1)	MCP-3(K)& MCP-5(K)	MCP-3 [87]: MCP-5: String [87],	IS	(816, − 1160)
MCP-1(K + 1)	$\overline{\mathrm{IL}-2}$ (K) & $\overline{\mathrm{MIP}-1\upbeta}$ MIP-1ϐ(K)	IL-2 [88, 89]: MIP-1β:String [85] [86],,	IS	(− 6150, − 6520)
MCP-3(K + 1)	IFN- γ (K) &MCP-1(K)	IFN- γ: String [90, 91], MCP-1: String [92],	IS	(−15,900, 16,800)
MCP-5(K + 1)	SCF(K) || $\overline{\mathrm{VEGF}-\mathrm{A}}$ (K)	SCF [93]: VEGF-A: KEGG	IS	(1300, − 1140)
OSM(K + 1)	MCP-1(K)	String [94, 95],	Output	1.11
SCF(K + 1)	FGF-9(K)* & IL-10(K)	IL-10 [96, 97]:	IS	(12,700, − 14,400)
TIMP-1(K + 1)	Lymphotactin*(K)	Not validated	Output	1.19
TNF-α(K + 1)	IL-2(K)	String [98, 99],	Output	−0.188
VEGF-A(K + 1)	MCP-5(K) & SCF(K)	MCP-5:String SCF: String [100, 101],	IS	(−13,400, 13,300)

**Table 6:** Boolean Functions and Description of IL-10 Stimulated M2c Activation Network. The * symbol denotes a non-validated link from public databases or literature

Child node	Parent node	References	Attribution	LP Weights
FGF-9(K + 1)	MIP-1β*(K)	Not Validated	IS	13
GM-CSF(K + 1)	$\overline{\mathrm{IP}-10}$ (K) & $\overline{\mathrm{IL}-2}$ (K) & IL-4(K) & IL-10(K)	IL-2: String [102], IL-4: String [102], [103] IL-10: [104, 105]	Output	(−0.83, − 0.73, 0.73, 0.75)
IFN-γ(K + 1)	IL-1α(K) & SCF(K)	IL-1α: String SCF: String	IS	(− 18,20)
IP-10(K + 1)	MCP-1(K) & MCP-3(K)	MCP-3: String MCP-1: String [106],	IS	(− 4470, 4050)
IL-1α(K + 1)	IL-11(K) & MCP-3(K)	IL-11: String MCP-3: String	IS	(− 699, 609)
IL-2(K + 1)	IL-10(K) & $\overline{\mathrm{IL}-11}$ (K)	IL-11: String IL-10: String,	IS	(5.62, −6.42)
IL-3(K + 1)	(FGF-9 (K) & $\overline{\mathrm{IL}-1\mathrm{a}}$ (K) & $\overline{\mathrm{MIP}-1}$ (K) & MCP-3(K)	FGF-9: String, MCP-3: [72] MCP-1: [107]	Output	(7.16, − 7.99, − 8.86, 8.68)
IL-4(K + 1)	IL-10(K)	String, [48, 108]	IS	41.6
IL-6(K + 1)	IL-1α(K) & MIP-1ϐ(K) & $\overline{\mathrm{MIP}-2}$ (K) & $\overline{\mathrm{VEGF}-\mathrm{A}}$ (K)	MIP-2: String, VEGF-A: String IL-1α: [45, 46] MIP-1ϐ [109, 110]:	Output	(2.26, 2.09, − 2.4, − 2.04)
IL-7(K + 1)	MCP-3(K)	String	Output	0.476
IL-10(K + 1)	MCP-3(K)	String	IS	3330
IL-11(K + 1)	IL-1α(K)	String [111, 112],	IS	−78.1
IL-12p70(K + 1)	MCP-3 (K) || $\overline{\mathrm{SCF}}$(K)	MCP-3 [113]: SCF: [114]	Output	(1.34, −1.38)
IL-17A(K + 1)	$\overline{\mathrm{MCP}-1}$ (K) || MCP-3(K)	MCP-1: String [115–118], MCP-3: String [80],	Output	(−0.236, 0.19)
Lympho-tactin(K + 1)	SCF(K)	String	IS	39
MIP-1β(K + 1)	IL-11(K)	String	IS	−16,100
MIP-2(K + 1)	SCF(K)	String [119],	IS	2420
MCP-1(K + 1)	MIP-1β(K) & SCF(K)	MIP-1β: String [85, 86], SCF: String [120, 121] [,^122^],	IS	(2050,1920)
MCP-3(K + 1)	IFN- γ (K)	IFN- γ: String [90, 91],	IS	1410
MCP-5(K + 1)	$\overline{\mathrm{SCF}}$ (K)	SCF [93]:	IS	−728
OSM(K + 1)	MIP-1β(K)	MIP-1β: String [123–125],	Output	0.186
SCF(K + 1)	IL-11(K) & MCP-5(K)	IL-11: String [126], MCP-5 [93]:	State	(14,700, − 18,000)
TNF-α(K + 1)	IP-10(K)	IP-10: String [127],	Output	0.156
VEGF-A(K + 1)	Lymphotactin (K) || $\overline{\mathrm{MCP}-5}$ (K)	MCP-5: String	Output	(19,200, −24,100)

M2a network has one input, IL-1α, which remains low consistently in the binarized profiles [71]. The M2c network has no input node. The shared output nodes for M2a and M2c include GM-CSF, IL-3, IL-6, IL-7, IL-12p70, IL-17, TNF-α, and OSM. IL-6 is an inflammatory cytokine and has demonstrated a consistently low profile in both M2 activation models [133, 134]. The low expression profile of IL-6 also indicates low expression profiles of Lymphotactin and IL-1a. TNF-α promotes the activation of inflammatory M1 cells and has a decreased value in M2 cells, which was also demonstrated by our M2 activation model [135, 136]. In addition, IL-6 and TNF-α, outputs of M2 macrophage, can reduce inflammation and kill the pathogen [134]. IL-12p70 is another common output that is elevated in M1 activation and remains as a logic low in both M2 models [137]. It has been reported that IL-17 induced the production of pro-inflammatory cytokines by human macrophage, but the quantitative analysis is still needed [138].

### Validation of IL-4 induced M2a and IL-10 induced M2c activation models

Parent and child nodes, Boolean functions, attribution of each node, interaction strengths from linear programming algorithm, and published results confirming the links in M2a and M2c networks were also shown in Tables 5 and 6, respectively. The majority of the links can be verified based on the KEGG pathway or protein interaction database.

The established M2a Boolean network includes a link between MIP-2 (Chemokine (C-X-C) motif ligand 2-CXCL-2) and FGF-9 which cannot be validated by STRING or KEGG database. The regulation between MIP-2 and FGF-9 is the most complicated Boolean function in all three activation networks. However, it has been reported that FGF-9 enhanced M2 differentiation post-myocardial infarction accompanying with significantly elevated IL-10 secretion [139]. MIP-2 as an inflammatory protein is typically secreted by M1 macrophages. Another un-validated link in the M2a network is inhibition of IL-7 by MCP-5. Since MCP-5 is typically expressed in M1 and IL-7 in M2 [10, 11, 13, 136, 140], we consider these as meaningful Boolean regulations and keep them in our network model. In addition, all these unvalidated links are links to output nodes in the network, which will not affect the core regulation. Once the expression profiles in the experiments agree with the Boolean function, the links are kept in the established M2a activation network.

The only un-validated link in the M2c network is MIP-1β to FGF-9. Again, the experimental results illustrated the effectiveness of the Boolean regulation and the link was kept in the network model. FGF-9 demonstrated elevated expression levels in three macrophage activation networks, while LPS stimulated macrophages produced much more FGF-9 than M2a and M2c [22].

M2 activation models were further validated by temporal transitions of their Boolean network models. M2a and M2c activation networks were simplified by removing the output nodes and the single directional signal transduction nodes. The core network for M2a and M2c activation networks are represented by proteins in diamond nodes in Figs. 8 and 9. Simplified M2a network contains a 7-protein core network including FGF-9, MCP-1, MCP-5, MIP-1β, MIP-2, SCF, and VEGF-A. This core network has multiple feedback loops including MCP-1 attractor, MIP-2 attractor, MCP-1 and MIP-1β interaction loop, MCP-5 and VEGF-A interaction loop, and VEGF-A and SCF interaction loop. Besides, there are feedback loops among VEGF-A, MIP-2, FGF-9, SCF, and MCP-5 (Additional file 1: Figure S2).

Simplified M2c network contains a 5-protein core network including IL-11, IL-1α, SCF, MCP-5, and IFN-γ as shown in Fig. 9. This core network has MCP-5 and SCF self-inhibition loop, IL-1α, and IL-11 interaction loop, IL-1a and IFN-γ interaction loop, and feedback loops among IL-1α, IL-11, SCF, IFN-γ. The link between IFN-γ and IL-1α was obtained by removing MCP-3 since MCP-3 is just a signal transduction state in this branch. Separated core networks for M1, M2a, and M2c activation were also available in (Additional file 1: Figure S1, S2, and S3). All these interaction loops were confirmed by previous studies or the KEGG pathway as shown in Table 6.

**Fig. 10:**
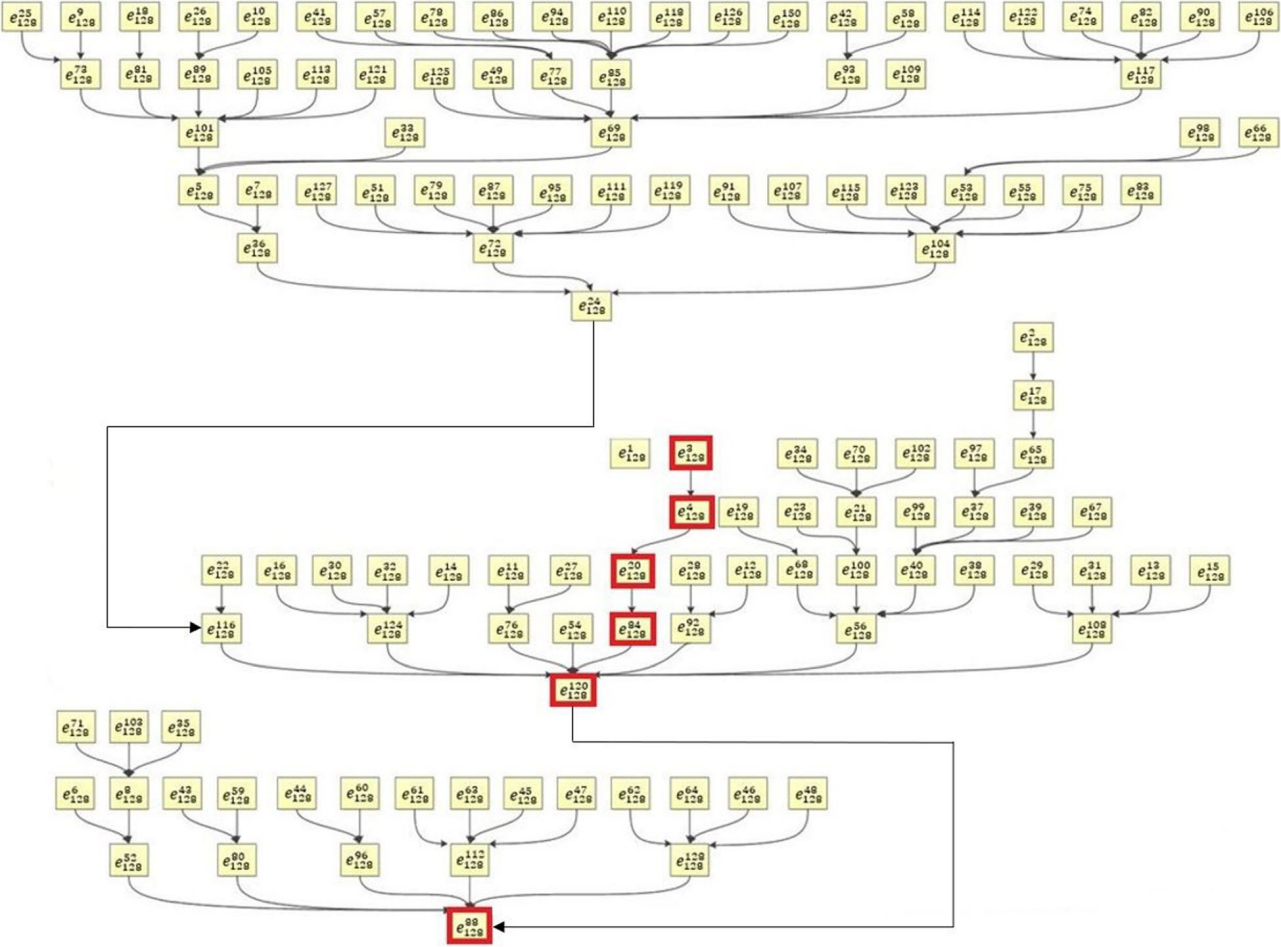
The evolution map of the core network for M2a activation showed all pathways predicted by the M2a activation Boolean network. The evolution of 128 (128 = 2^7^) states for 7 nodes in the core network were illustrated from different initial conditions. The states in red boxes represented the evolution path based on the temporal cytokine profiles in this study

**Fig. 11:**
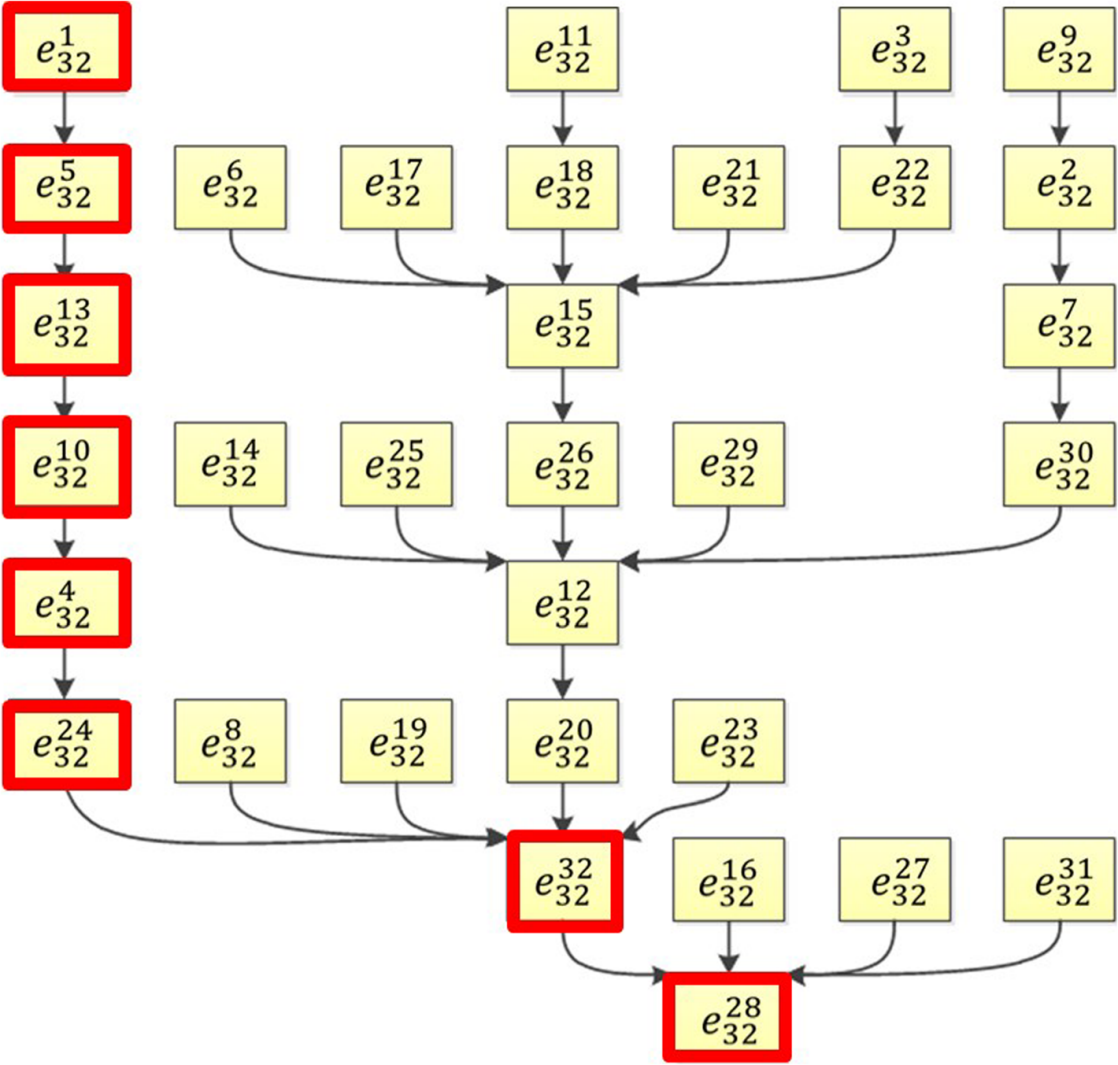
The evolution map of the core network for M2c activation showed all pathways predicted by the M2c activation Boolean network. The evolution of 32 (32 = 2^5^) states for 5 nodes in the core network were illustrated from different initial conditions. The states in red boxes represented the evolution path based on the temporal cytokine profiles in this study

Evolutionary maps of M2a and M2c were shown in Fig. 10 and Fig. 11, respectively. The evolution of M2a has a state transition map that contains 128 = 2^7^ states while M2c has a state transition map for 32 = 2^5^ states. The binarized M2a temporal profiles in our dataset showed the transition from ${e}_{128}^3\to {e}_{128}^4\to {e}_{128}^{20}\to {e}_{128}^{84}\to {e}_{128}^{120}$ and these states were illustrated in red boxes in Fig. 10. The binary values of these 7 states were shown in Table 7. M2c network has binarized transitions from ${e}_{32}^1\to {e}_{32}^5\to {e}_{32}^{13}\to {e}_{32}^{10}\to {e}_{32}^4\to {e}_{32}^{24}\to {e}_{32}^{32}\to {e}_{32}^{28}$ illustrated in red boxes in Fig. 11. The binary values of these 5 states were shown in Table 8. The computational binary state transition was compared with temporal experimental profiles and converged to experimental results.

**Table 7:** Binary Expressions of Proteins in the Core Network for IL-4 Stimulated Macrophage Activation. The binarization is shown in two’s complement representation

State	VEGF-A	FGF-9	MIP-1β	MCP-5	SCF	MIP-2	MCP-1
${e}_{128}^3$	1	1	1	1	1	0	1
${e}_{128}^4$	1	1	1	1	1	0	0
${e}_{128}^{20}$	1	1	0	1	1	0	0
${e}_{128}^{84}$	0	1	0	1	1	0	0
${e}_{128}^{120}$	0	0	0	1	0	0	0
${e}_{128}^{88}$	0	1	0	1	0	0	0

**Table 8:** Binary Expressions of Proteins in the Core Network for IL-10 Stimulated Macrophage Activation. The binarization is shown in two’s complement representation

State	IL-11	SCF	MCP-5	IL-1α	IFN-γ
${e}_{32}^1$	1	1	1	1	1
${e}_{32}^5$	1	1	0	1	1
${e}_{32}^{13}$	1	0	0	1	1
${e}_{32}^{10}$	1	0	1	1	0
${e}_{32}^4$	1	1	1	0	0
${e}_{32}^{24}$	0	1	0	0	0
${e}_{32}^{32}$	0	0	0	0	0
${e}_{32}^{28}$	0	0	1	0	0

## Discussion

The most significant contribution of this study includes the following facts. We established and validated novel Boolean networks for 3 macrophage activation patterns using in vitro temporal cytokine profiles. Our results confirmed 1 stable M1 macrophage activation state and 2 stable M2 activation states, which agreed with reported in vitro classification on macrophage activation patterns. Validation of the models was conducted based on the coincidence of predicted cytokine expression levels, public databases, and reported experimental results. The literature review confirmed the majority of links in the Boolean networks for M1, M2a, and M2c network models as shown in Tables 3, 5, and 6, respectively. More importantly, our results demonstrated possible key factors IFN-γ, IL-1α, Lymphotactin, MCP-1, MCP-5, and SCF for M1 activation, FGF-9, MCP-1, MCP-5, MIP-1β, MIP-2, SCF, and VEGF-A for M2a activation IFN-γ, IL-1α, IL-11, MCP-5, and SCF for M2c activation. Further, expression profiles of these cytokines can serve as signatures for macrophage activation patterns since other protein expressions can be determined by proteins in the core network and input proteins of the network. These core signatures provide a possible mixed combination for macrophage activation markers instead of using only static individual markers. Additionally, cytokine markers might be more useful for in vivo study for easy and less costly measurements.

Robustness of Boolean networks is an important research aspect while only a few results have been reported [141–143]. These results have shown a large basin for the robustness and stability of a Boolean network. In this study, the simulated biggest core network for M1 macrophage activation has two stable states: $$ {e}_{64}^7 $$ and $$ {e}_{64}^{58} $$ as shown in Fig. 6. The state $$ {e}_{64}^7 $$ represents a state when MCP-5, Lymphotactin, IL-1α, and MCP-1 have elevated expression levels, SCF and IFN-γ have decreased expression levels. This is a single state attractor meaning the Boolean network starting from this initial state will stay here forever. All of the other 63 states of the network will evolve to the state of $$ {e}_{64}^{58} $$, which is a controversial state of $$ {e}_{64}^7 $$ (SCF and IFN-γ are elevated and other 4 proteins are down-regulated). Similarly, the core network for M2a has two stable states $$ {e}_{128}^{88} $$, and $$ {e}_{128}^1 $$ (Fig. 10). In the state of $$ {e}_{128}^1, $$ all 7 proteins have elevated expression levels. The core network of M2c activation has only one stable state $$ {e}_{32}^{28} $$ as shown in Fig. 11. All these have demonstrated the robustness of the established Boolean networks.

The STRING PPI database integrates several PPI databases and is the most widely adopted database for protein-protein interactions. We only use the STRING PPI database to confirm possible interactions among proteins in the established Boolean networks. Since the STRING PPI database does not provide directional information for protein-protein interactions, the directional information of our Boolean networks is generated by the linear programming algorithm computationally. The confirmation of directions in the network can be indirectly confirmed by 1) their positions in up- or downstream of pathways since cytokines can be either stimuli or production of a pathway; and 2) the temporal sequence of significantly elevated or inhibited expressions in biological experiments.

## Conclusion

The proposed method can also be applied to large scale datasets to establish regulatory Boolean networks for other biological processes. Due to the limited sampling time points in most of the deposited experimental datasets, Boolean networks might be much more promising than traditional differential equation models, which require determination and estimation on a huge amount of parameters. In addition, with measurements at limited time points, deep learning might not be a good approach to attack specific biological processes at the current stage. It’s worth to mention, this method can be applied to a small set of key factors extracted from big data, and the following computational prediction and validation of the small networks are much easier than the big network. Currently, our software package (Matlab) can easily handle the evolution of a Boolean network with 11 nodes, leading to a total of 2^11^ transition states using a normal workstation. For larger Boolean networks with more than 11 nodes, high-performance computing is more suitable.

We did notice some limitations of the proposed modeling method due to noisy/outlier functions. Outliers can still cause the linear programming to gather an extra connection, drop a connection or give a wrong sign to a connection. Additionally, a time delay can also cause problems in determining Boolean regulations depending on the amount of delay. For example, if protein A promotes protein B in a motif, up-regulation of protein A should give an instant high expression of protein B in the mathematical model. However, time delays and variations of temporal responses are very common in biological responses. One of our modeling assumptions is the Boolean network is fixed without changing nodes and changing Boolean functions in the response time period. Accordingly, Boolean functions are determined with respect to the whole experimental time period, not the single time point In this study. Therefore, small time-delays or variations of temporal response may affect the Boolean regulation at a short period of time, but won’t affect the Boolean regulation in the whole time period. However, if experimental results have a long delay on the up-regulation of protein B, incorrect Boolean functions may be selected. Finally, the model will be limited to the proteins that were measured in the experiment. Any other proteins not sampled were left out. Further studies will be conducted to integrate cross-platform data for a more complete macrophage activation model in the future.

Three Boolean network models were established to elucidate the regulation of M1, M2a, and M2c macrophage activations. Prediction of the Boolean network models agree with experimental results and validated the established models. Signatures of core cytokine profiles were determined, which provided a possible examination for macrophage activations based only on the cell productions.

## Methods

Data Preparation: A total of 27 cytokines’ expression levels from macrophages isolated from C57BL/6 J male mice (Jackson Labs, Bar Harbor, ME) were collected at 7 time points, setpoint (0 h, 0 h), 0.5 h, 1 h, 3 h, 6 h, 12 h, 24 h from 4 biological groups (4 replicates/group) including 1 control group and 3 macrophage polarized groups: M1 induced by LPS, M2a induced by interleukin 4 (IL-4), and M2c induced by interleukin 10(IL-10) [22].

All proteins in the established networks are cytokines, which are small, nonstructural proteins, including interleukins, chemokines, interferons, and tumor necrosis factors [144]. A total of 34 cytokines have been reported in the literature and are secreted by different cell types including macrophages, T-cells, neutrophils, and fibroblasts. In the manuscript by Melton’s group, 24 proteins were reported. IL-1α, IL-6, and TNF-α were measured in their experiments and included in their dataset, but not reported because the authors thought these three cytokines were related to M2b activation [22]. All 27 cytokines secreted by macrophages were used to establish the Boolean network model in this study.

Due to the limited temporal measurements, a curve fitting algorithm was applied to enrich the dataset and this dataset served as inputs for a linear programming algorithm to find the most significant protein (node) and connections (links) for network structure. The same enriched data was also analyzed by an iterative K-means method to find the threshold for binarization. Thus, a network could be established where each node represents a protein and each link between the nodes represents an association of two proteins. Meanwhile, the expression level of each node was defined as a Boolean variable ‘0’ or ‘1’. A Karnaugh map was used to determine the Boolean regulatory functions among these nodes/variables to create a Boolean network model. Verification of the regulations was conducted based on published interactions from PPI databases and literature searches. The predicted evolution of each Boolean network model for a macrophage activation pattern was further validated with public databases and published results.

### Curve fitting

The smooth spine algorithm in Matlab was applied to approximate continuous secretion profiles for 27 proteins. The algorithm was chosen due to its ability to construct an accurate curve with noisy data. By minimizing the squared error between the constructed data and experimental data at the sampling time points, optimized fitting curvature was obtained using the following equation [145]:

1 $$ p{\sum}_i{a}_i{\left({x}_i-s\left({t}_i\right)\right)}^2+\left(1-p\right)\int {\left(\frac{d^2s}{d{t}^2}\right)}^2 dt, $$

where *p* denotes the smoothing parameter controlling the level between the smoothness of the function and the error between the experimental data *x*_*i*_ and the value of the fitting function *s*(*t*_*i*_) at chosen time points *t*_*i*_. Parameter *a*_*i*_ represents the weight of the error, which is set to be the default value in MATLAB. Once the continuous fitting function *s*(*t*_*i*_) was gathered, we divided the 24-h time span into 50-time points with 40 being evenly sampled from the initial reading to the 9th hour and the last 10 samples coming from the 9th hour to the final 24. This was set because the majority of regulations occurred within the first few hours [19–22]. No negative value is allowed in the fitting algorithm to match with real biological situations.

### Binarization

In a Boolean network model, protein expression is either up-regulated or down-regulated, therefore, binarization of the fitting function is needed. Any protein with similar expression levels during the 24 h time period is considered non-significant and will not be modeled. The iterative K-means method was used to cluster the expression levels for each protein and determine an activation threshold for each stimulus and experiment [146, 147]. The objective function for iterative K-means method using the absolute difference from the point to the center of the cluster was defined as:

2 $$ J={\sum}_{j=1}^k{\sum}_{i=1}^n\mid {x}_i-{C}_j\mid, $$

Where *k* is the cluster number, *C*_*j*_ is the centroid of the *j*_*th*_ cluster, *i* represents each sample, and *n* is the total number of samples (50 in this study). We started with 8 clusters for iterative K-Means method and reduced to 2 clusters. The average of the mean distance from each cluster was used as the activation threshold for a specific protein. Any expression level below the activation threshold is defined as “0” and above the threshold is defined as “1” for binarization.

The threshold could be a value of 0 if there was no reading at any of the time points, or no significant change in protein expression levels. However, if a threshold of 0 will be considered in our modeling procedure, any non-zero low expression measurement will be considered active. To avoid such a conflict, a threshold of value “0” was changed to a low value claimed by the detection range of the biological experiment, such as 1 × 10^− 6^ in this study.

### Linear programming algorithm

A network with connections among 27 proteins and each interaction strength was determined by linear programming method with an R package [148, 149]. In the network model, each protein and interaction among proteins is represented as a node with a link. Each node is assigned with an activity level *x*_*i*_ *ϵ R*^*+*^ which is the expression level of each protein. Each link is assigned a weight, *w*_*j*, *i*_, form parent node *j* to the child node *i*. The weights *w*_*j*, *i*_ *ϵ R* with *w*_*j*, *i*_ *> 0* representing promotion and *w*_*j,I*_ *< 0* for inhibition. Also, each protein has an activation threshold, *δ*_*i*,_ determined by the Binarization method above. The activity level of each node *x*_*i*_ can be determined by its initial status ${w}_i^0$, the activity level of a parent node *x*_*j*_ and contribution weight *w*_*j*, *i*_ from the parent node as

3 $$ {x}_i={w}_i^0+{\sum}_{j\ne i}{w}_{j,i}{x}_j, $$

Considering biological networks are sparse, we minimize the sum of the absolute link weights $\sum \limits_{i,j}\mid {w}_{j,i}\mid$, the bias term $\sum {w}_i^0$, and noise effects denoted by *ζ*_*l*_. A random variable *ζ*_*l*_*ϵ R*_*0*_^*+,*^ *l = 1., …, L,* is introduced to accounts for data variations in the experimental data. L represents the set of inactive proteins in the experimental data and coincides with the number of constraints that might violate the linear programming. The objective function was defined as:

4 $$ \mathit{\min}{\sum}_{i,j}\mid {w}_{j,i}\mid {x}_j+\frac{1}{Y}{\sum}_i{w}_i^0+\frac{1}{\lambda }{\sum}_l{\upzeta}_{\mathrm{l}} $$

subject to the constraints of

5 $$ \forall \left(i,m\right)\  where\ {x}_{i,m}\ge {\delta}_i:{w}_i^0+{\sum}_{j\ne i}{w}_{i,j}{x}_{j,m}\ge {\delta}_{i,m} $$

6 $$ \begin{aligned} \forall \left(i,m\right)\  where\ {x}_{i,m}&\ge {\delta}_i:{w}_i^0+{\sum}_{j\ne i}{w}_{i,j}{x}_{j,m}\\
&\ge 0 + {\upzeta}_{\mathrm{l}} \end{aligned}$$

where the constraints are defined for each experiment performed, *mϵ* 1,2,3,4, representing the 4 replicates in each experimental group, and for each time point from the enriched fitting data. Constraint (5) selected proteins up-regulating specific protein while constraint (6) looks for proteins down-regulating the protein. Effects of the data variation are minimized by the term $\frac{1}{\lambda}\sum \limits_l{\upzeta}_{\mathrm{l}}$ in the objective function (4).

In the objective function, the production term, |*w*_*j*, *i*_|*x*_*j*_ shows the contribution to the child node expression *x*_*i*_ level from the parent node *x*_*j*_.Two parameters γ and λ are non-negative weights of the bias term and slack variables representing the penalties on the initial values and data variations in the objective function. For an ideal parameter, *λ*, 5-fold cross-validation was used with the smallest mean square error limiting the value of *λ* a maximum set of *Lσ*^*2*^*(x*_*j,m*_*)*, where *σ* is the variance of a variable. Parameter *Υ* in the objective function (4) represents the weights on the bias ${w}_i^0$. A bigger value of *Υ* (*Υ* = 100) in this study will put more penalty on the link of the proteins rather than bias. Minimizing this term helps to find the most significant connection to the child node, *x*_*i*._

We further modified the linear programming algorithm to simplify the network structure. For each child node *x*_*i*_, a weighted contribution |*w*_*j*, *i*_|*x*_*j*_ from a parent node (*x*_*j*_) is calculated. The maximum value of all weighted contribution from a parent node can be determined. For any node, if a value of |*w*_*j*, *i*_|*x*_*j*_ is larger than 70% of the maximum value related to the node, the link from node *j* to node *i* is kept; In addition, orphan nodes and a node without significant changes in expression levels were not included in our model. The final output of the modified linear programming is a set of nodes and links with interaction strength *w*_*j*, *i*_. Positive *w*_*j*, *i*_ means promotion while negative *w*_*j*, *i*_ means inhibition.

### Establish the Boolean logic function

Theoretically, to determine a Boolean regulation with 3 nodes, 8 (2^3^) states representing the status of the 3 nodes are needed. It means at least 8-time points are needed and each time point represents a unique status of the network. Since we only have data at 7-time points, curving fitting with respect to time and resample the fitted data at different time points is reasonable to enrich the dataset. The 50 samples generated from the fitted data allows us to determine a Boolean function for up to 5 nodes ideally since 2^5^ = 32 < 50. Considering the same status of the system may occur at different time points and most biological sub-networks have dual or triplet nodes, a maximum 5-node sub-network motif is expected with 50-time points in this study. It’s worth to mention, that the number of time points to be selected depends on how fast the dynamics evolve and the complexity of the network, is not the focus of this study and won’t be discussed in detail.

Based on assigning expression levels ‘0’ or ‘1’ to each node, a truth table of a motif including parents and child nodes can be established. Boolean regulation between parent and child node was determined by the Karnaugh map.

If there is a mismatch of promotion or inhibition between Boolean relation obtained from Karnaugh Map and linear programming or experimental profiles, then an extended 200 samples were re-examined (50 from each replicate and 4 replicates in each group) to determine the Boolean function instead of using the average. This can occur when one set of data has a large variation from the other 3 replicates, causing a skew in the average or when a time delay affects promotion and inhibition responses. The 200-sample-set was also used to determine if self-regulation could have been an option, which was mainly done on the results that always remained logic high, occurring once in the M1 cell and validated with experimental results.

### Mathematical modeling and validation

**Fig. 12:**
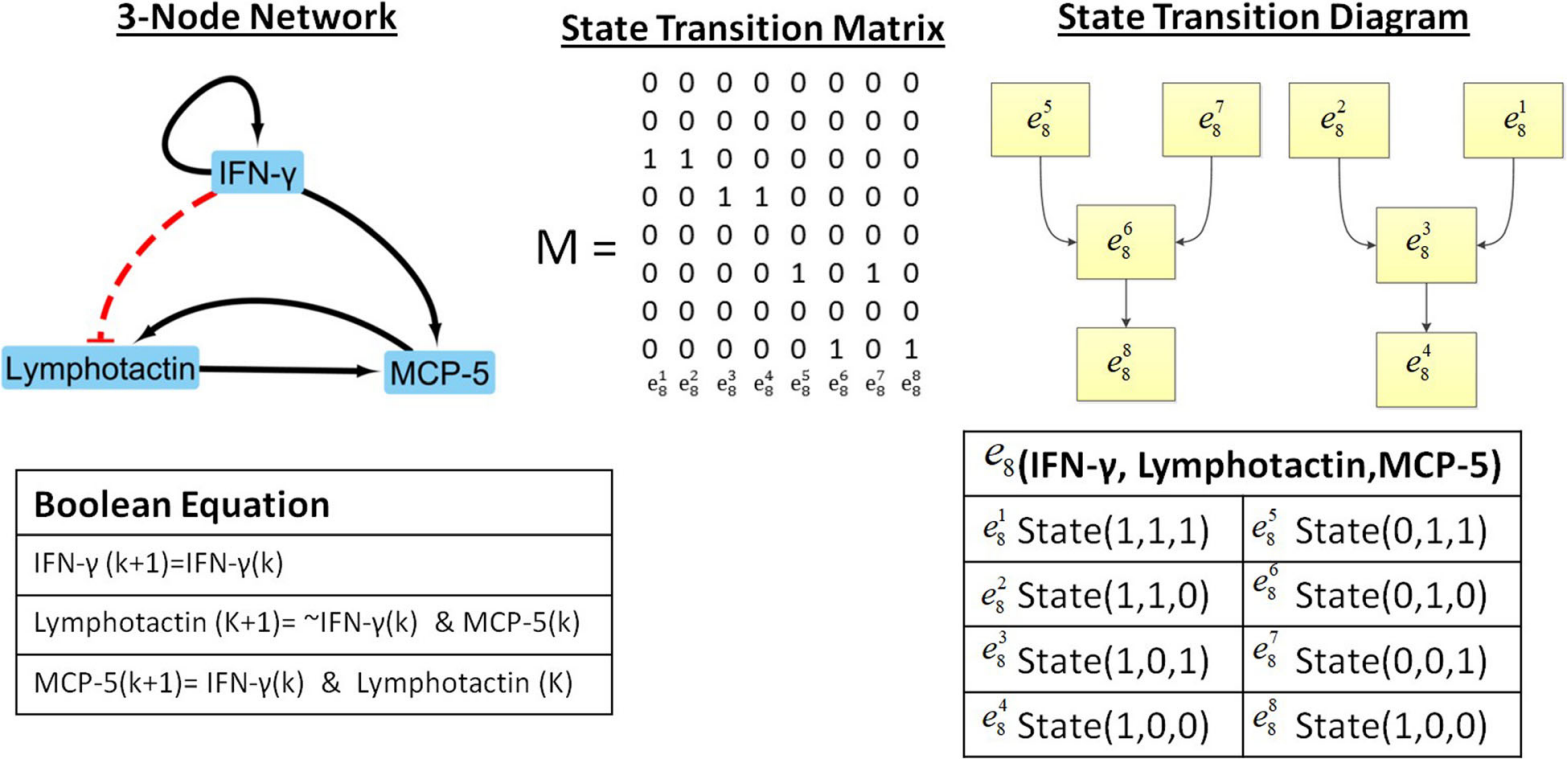
The Boolean functions and evolutionary map for a 3-node network were illustrated

The linear programming approach determined the nodes and possible connections in a network. Boolean regulations among nodes were determined by the Karnaugh map and represented by Boolean functions. With each node in the network as a Boolean variable *X*, the evolution of the status of a network can be written as:

7 $$ X\left(k+1\right)=M\ltimes X(k), $$

where *k* represents current time point and *k* +1 for next time point, *X* (*k*) and *X* (*k* + 1) represent current and future states of the network, *M* is the state transition matrix determined by Boolean functions and ⋉ represents the semi-tensor product [150]. We and other research groups have established computational tools for Boolean network models that are available for free download [150–152]. .An example of determining a 3-node network is shown in Fig. 12. The 8 states, state transition matrix, and evolution of the 8 states for the 3-node network was computed with our own software package [151]. All codes associated with macrophage polarization can be found in our GitHub link (https://github.com/RicardoRamirez2020/Macrophage_Boolean_Network_LP).

### Establishment of a core network

Dynamics of Boolean networks become more complicated with increasing numbers of nodes and Boolean functions, resulting in difficulties in understanding and analysis of the network. Given a network with N nodes, there exist 2^*N*^ states of the network and the state transition matrix will be 2^*N*^ by 2^*N*^ dimension, indicating a huge computational cost. Simplification of a Boolean network for easy analysis has been conducted to reduce both the number of nodes and functions while still keeping the capability of predicting all states of the network. It’s worth to mention, output nodes of a network only have incoming signals and do not affect the regulations of the network. In addition, if multiple nodes are connected by single direction links, all intermediate nodes only transduce the signal with promotion (keep the signal) or inhibition (Not operation). These nodes with single direction connections can be simplified by keeping the starting and ending nodes and the corresponding Boolean function can be derived with basic logic operations. Therefore, by removing the output and intermediate transduction nodes, a core network can be established to represent a Boolean network with much simpler Boolean functions and fewer states for simulation. Meanwhile, with the determined states of a core network, states of removed nodes can be reconstructed.

## Supplementary information

**Additional file 1: Figure S1.** The core network for M1 activation by removing output nodes and signal transduction node. **Figure S2.** The core network for M2a activation by removing output nodes and signal transduction node. **Figure S3.** The core network for M2c activation by removing output nodes and signal transduction node.

## Data Availability

The datasets used and analysed during the current study are available from the corresponding author on reasonable request.

## Abbreviations

FGF-9: Fiboblast Factor 9
GM-CSF: Granulocyte-Macrophage Colony-Stimulating Factor
IC: Immune Complex
IFN-γ: Interferon Gamma
IL-10: Interleukin-10
IL-11: Interleukin-11
IL-12p70: Interleukin-12, p70
IL-17A: Interleukin-17A
IL-1α: Interleukin-1 Alpha
IL-2: Interleukin-2
IL-3: Interleukin-3
IL-4: Interleukin-4
IL-6: Interleukin-6
IL-7: Interleukin-7
IP-10: (CXCL10) C-X-C Motif Chemokine Ligand 10
KC-GRO: (CXCL1) – C-X-C Motif Chemokine Ligand 1
LP: Linear Programming
LPS: Lipopolysaccharide
M1: Classically Activated Macrophages
M2: Alternatively Activated Macrophages
MCP-1: (CCL2) Monocyte Chemoattractant Protein 1
MCP-3: (CCL7) Monocyte Chemoattractant Protein 3
MCP-5: (CCL12) Monocyte Chemoattractant Protein 5
MIP-1β: (CCL4) C-C motif Chemokine Ligand 4
MIP-2: (CXCL2) C-X-C motif Chemokine Ligand 2
mRNA: Messenger Ribonucleic Acid
OSM: Oncostatin M
PPI: Protein to Protein Interactions
RANTES: (CCL5) Regulated on Activation Normal T Cell Expressed and Secreted
SCF: Stem Cell Factor
TGF- β: Tumor Growth Factor Beta
TIMP-1: Tissue Inhibitor of Metalloproteinases
TLR: Toll-Like Receptor
TNF-α: Tumour Necrosis Factor Alpha
VEGF-A: Vascular Endothelial Growth Factor A
