# Supplementary material for: Deriving a Boolean dynamics to reveal macrophage activation with in vitro temporal cytokine expression profiles
Source: BMC Bioinformatics. 2019 Dec 18;20:725. doi: 10.1186/s12859-019-3304-5 (PMC6921543; doi:10.1186/s12859-019-3304-5)

Figure S1. The core network for M1 activation by removing output nodes and signal transduction node. Status of these 6 proteins is determined by their initial conditions and regulations among them. Status of other cytokines in M1 activation network can be determined based on the status of these 6 proteins.


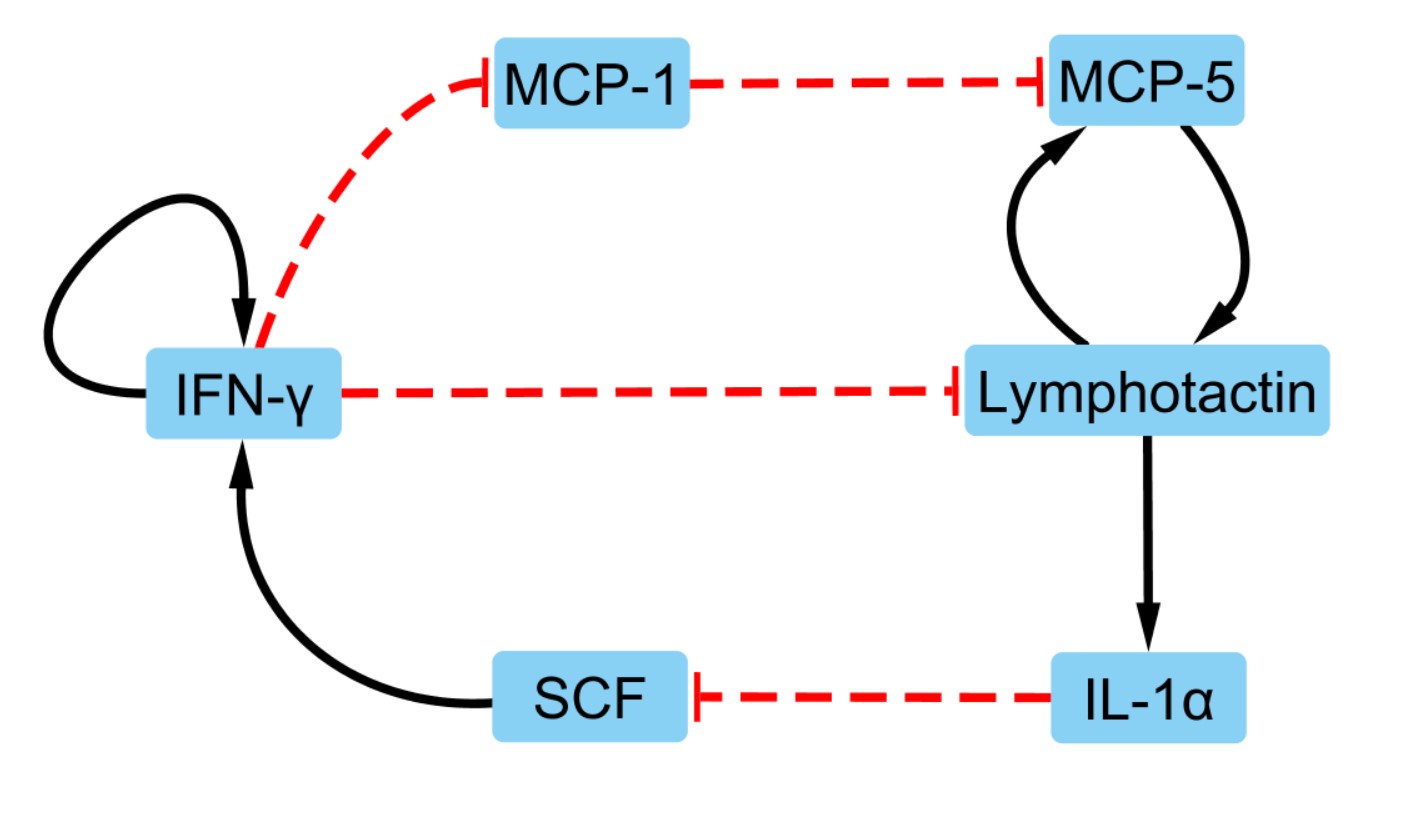


Figure S2. The core network for M2a activation by removing output nodes and signal transduction node. Status of these 7 proteins is determined by their initial conditions and regulations among them. Status of other cytokines in M1 activation network can be determined based on the status of these 7 proteins.


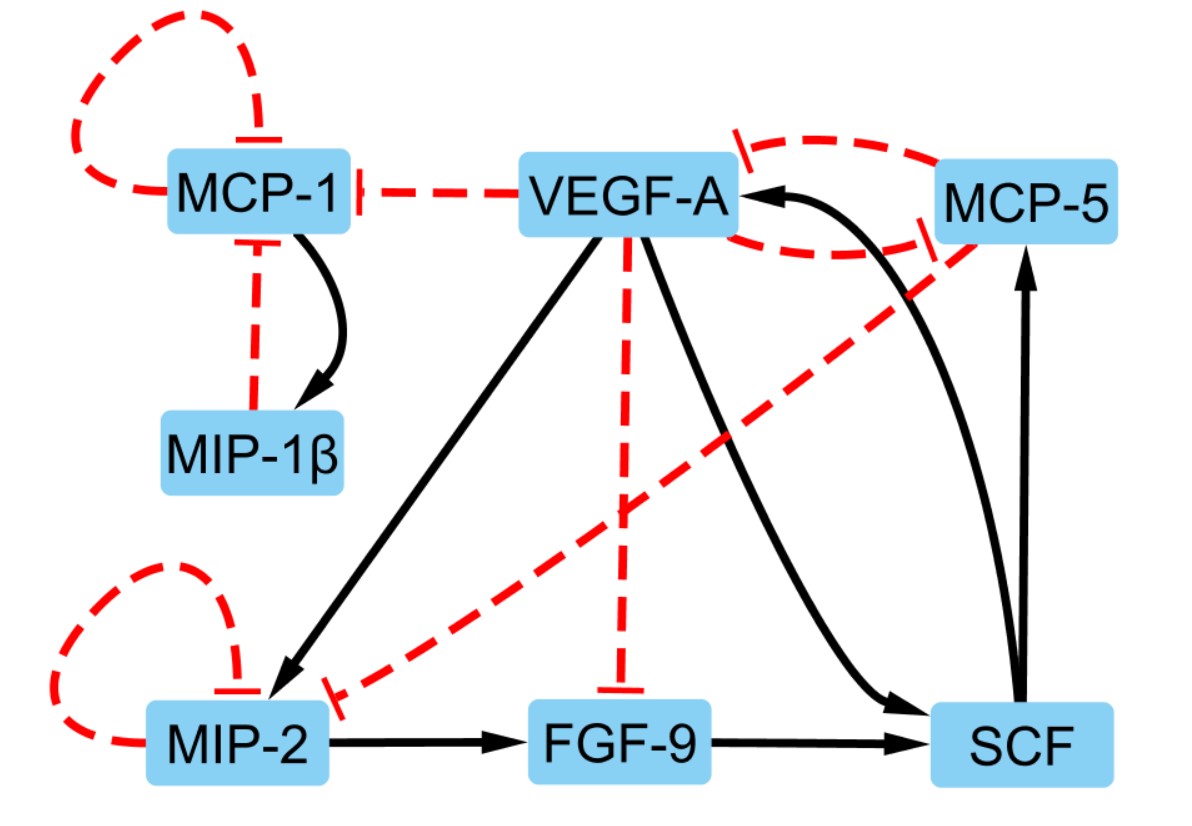


Figure S3. The core network for M2c activation by removing output nodes and signal transduction node. Status of these 5 proteins is determined by their initial conditions and regulations among them. Status of other cytokines in M2c activation network can be determined based on the status of these 5 proteins.


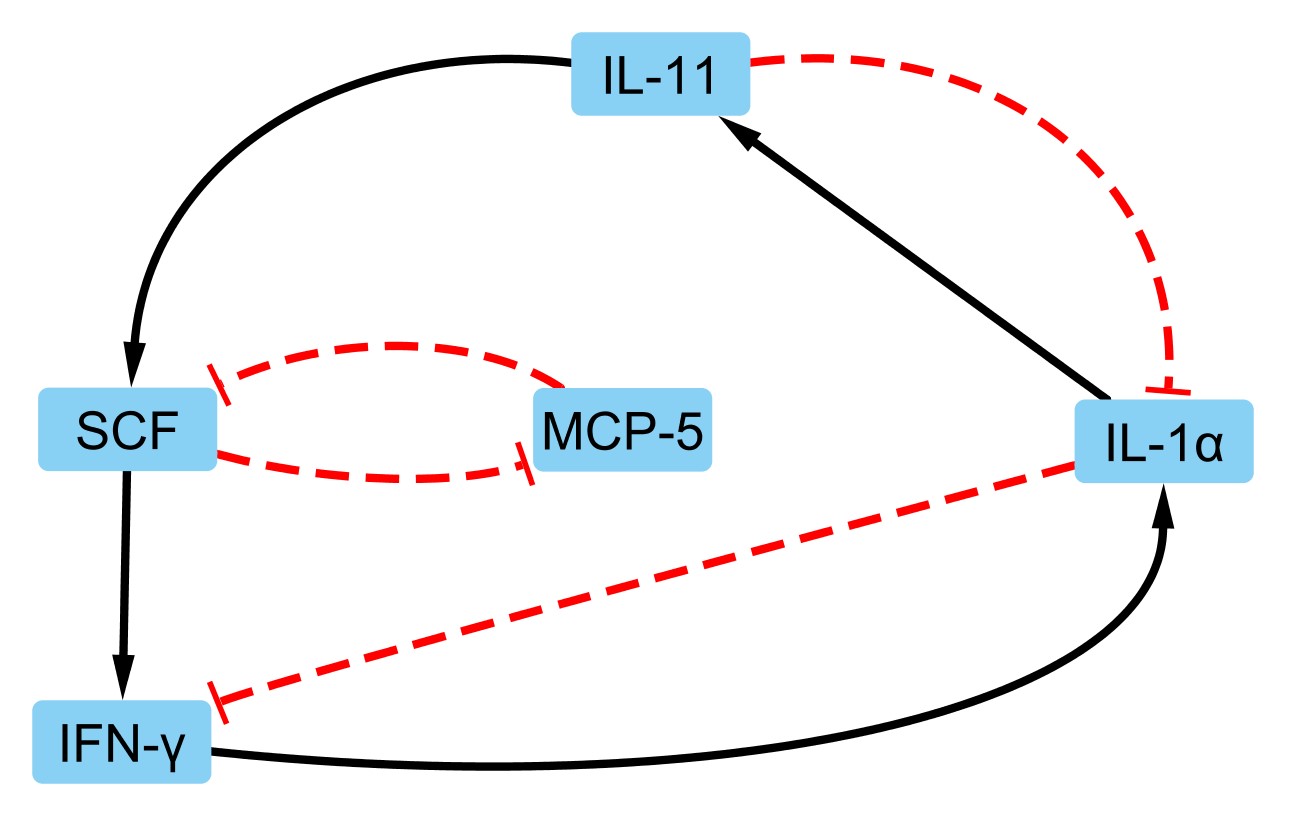

Supplement: Supplementary file 1 — Additional file 1: Figure S1. The core network for M1 activation by removing output nodes and signal transduction node. Figure S2. The core network for M2a activation by removing output nodes and signal transduction node. Figure S3. The core network for M2c activation by removing output nodes and signal transduction node. [file 12859_2019_3304_MOESM1_ESM.docx]
